# Evolutionarily Conserved Substrate Substructures for Automated Annotation of Enzyme Superfamilies

**DOI:** 10.1371/journal.pcbi.1000142

**Published:** 2008-08-01

**Authors:** Ranyee A. Chiang, Andrej Sali, Patricia C. Babbitt

**Affiliations:** Departments of Biopharmaceutical Sciences and Pharmaceutical Chemistry and California Institute for Quantitative Biosciences, University of California at San Francisco, San Francisco, California, United States of America; European Molecular Biology Laboratory, Germany

## Abstract

The evolution of enzymes affects how well a species can adapt to new environmental conditions. During enzyme evolution, certain aspects of molecular function are conserved while other aspects can vary. Aspects of function that are more difficult to change or that need to be reused in multiple contexts are often conserved, while those that vary may indicate functions that are more easily changed or that are no longer required. In analogy to the study of conservation patterns in enzyme sequences and structures, we have examined the patterns of conservation and variation in enzyme function by analyzing graph isomorphisms among enzyme substrates of a large number of enzyme superfamilies. This systematic analysis of substrate substructures establishes the conservation patterns that typify individual superfamilies. Specifically, we determined the chemical substructures that are conserved among all known substrates of a superfamily and the substructures that are reacting in these substrates and then examined the relationship between the two. Across the 42 superfamilies that were analyzed, substantial variation was found in how much of the conserved substructure is reacting, suggesting that superfamilies may not be easily grouped into discrete and separable categories. Instead, our results suggest that many superfamilies may need to be treated individually for analyses of evolution, function prediction, and guiding enzyme engineering strategies. Annotating superfamilies with these conserved and reacting substructure patterns provides information that is orthogonal to information provided by studies of conservation in superfamily sequences and structures, thereby improving the precision with which we can predict the functions of enzymes of unknown function and direct studies in enzyme engineering. Because the method is automated, it is suitable for large-scale characterization and comparison of fundamental functional capabilities of both characterized and uncharacterized enzyme superfamilies.

## Author Summary

Enzymes are biological molecules essential for catalyzing the chemical reactions in living systems, allowing organisms to convert nutrients into usable forms and convert harmful or unneeded molecules into forms that can be reused or excreted. During enzyme evolution, enzymes maintain the ability to perform some aspects of their function while other aspects change to accommodate changing environmental conditions. In analogy to studies of enzyme evolution focused on conservation of sequence and structural motifs, we have examined a large number of enzyme superfamilies using a new computational analysis of patterns of substrate conservation. The results provide a more nuanced picture of enzyme evolution than obtained either by detailed small-scale studies or by large-scale studies that have provided only general descriptions of function and substrate similarity. The superfamilies in our set fall along the entire spectrum from the conserved substructure being mostly reacting to mostly nonreacting, with most superfamilies falling in the intermediate range. This view of enzyme evolution suggests more complex patterns of functional divergence than those that have been proposed by previous theories of enzyme evolution. The method has been automated to facilitate large-scale annotation of enzymes discovered in sequencing and structural genomics projects.

## Introduction

The molecular functions of enzymes result from a complex evolutionary interplay between environmental constraints, requirements for organismal fitness, and the functional malleability of a particular enzyme scaffold. Within these constraints, existing enzymes are recruited during evolution to perform new or modified functions while often maintaining some aspects of the ancestral function [1]–[3]. Consequently, among contemporary enzymes we observe groups of evolutionarily related enzymes that share some aspects of molecular function and differ in others. The most divergent groups of evolutionarily related enzymes that still share aspects of function are called superfamilies. Within a superfamily, we define a family as a set of proteins that perform the same overall catalytic reaction in the same way. Why are some aspects of function shared and others allowed to change? By examining which aspects of function are shared among contemporary enzymes, we can gain insight into the requirements and constraints that govern this evolutionary process.

The focus of most studies of enzyme evolution has been the examination of conservation in sequence and structure. The data available to conduct such studies is enormous and still increasing due to the multiplicity of ongoing genomic and metagenomic sequencing efforts [4]. In tandem with the growth of sequence and structural data, a large number of new and sophisticated tools have been developed to improve our ability to identify the divergent members of superfamilies, allowing us to analyze patterns of conservation in sequence and structure that shed light on how enzyme functions have evolved and diversified (for some examples, see [5]–[7]). But such studies only capture aspects of enzyme evolution that can be inferred from the machinery that enables enzymatic catalysis, the enzymes themselves. Far fewer studies have focused on the substrates and products of these reactions, with most of these focused on the requirements of metabolism [8],[9]. In this work, our goal is to understand the details of how enzymes function and evolve by studying the conservation and variation in their substrates and products. In doing so, we aim for a more extensive view of enzyme evolution in order to improve our abilities to annotate enzymes of unknown function and to infer common aspects of function for superfamilies that have not yet been characterized.

The value of any analysis of the evolution of enzyme function depends on how we describe enzyme function, with respect to both the detailed molecular functions of individual enzymes and the properties of function shared across diverse members of enzyme superfamilies. Previous approaches to study enzyme evolution range from detailed manual analyses of small numbers of related enzyme families and superfamilies to automated analyses of many superfamilies. The former have often included not only analyses of sequences and structures but also comparisons of the substrates and reaction mechanisms of the constituent enzymes. These studies have been useful for annotating new sequences and structures and for generating and testing hypotheses about patterns of enzyme evolution (see [10]–[14] for examples). However, because of the expert knowledge required and their time-intensive nature, these types of analyses are not feasible for large numbers of superfamilies. Other semi-automated efforts have contributed to our understanding of enzyme evolution and data from these analyses have been made available in a number of online resources that include the Structure-Function Linkage Database [15], MACiE [16], the Catalytic Site Atlas [17], and EzCatDB [18]. Automated analyses more directly comparable to the large-scale and automated study described here [19]–[21] have used enzyme classification systems, like the Enzyme Commission (EC) system [22], to represent functional properties and determine what properties are conserved. The EC system represents a large proportion of known enzyme reactions, classifying each enzyme with a hierarchical set of four numbers that uniquely identify a reaction, and is easy to use for large-scale analyses. However, this system, developed before analyses of enzyme evolution were common, does not provide a detailed description of enzyme function or substrates at the atomic level [23]. Moreover, the EC classification of function often does not correspond with either the aspects of function that are conserved or those that can change during evolution. These issues make this system unsuitable for evaluating how enzyme function evolves, especially when evolutionary relationships are distant [24]. For enzymes, the Gene Ontology (GO) system's [25] molecular function classifications, also often used to describe and analyze function, largely recapitulate the EC system. More similar to the work reported here, several groups have analyzed enzyme relationships and evolution using substrate and reaction similarities [26]–[28]. Although these similarity metrics are useful, especially for clustering enzymes by their substrate similarities, they are not informative about what specific aspects of function are conserved, a specific goal of this work.

Here, we use graph isomorphism analyses to compare substrates of enzymes from 42 superfamilies to identify specific aspects of function conserved within each superfamily. We also use comparisons of substrates and their corresponding products to determine whether and how much of the conserved substructure is involved in the reaction. This comparison of substrates and products is similar to an analysis performed for a previous study with a different purpose, to predict EC numbers [29]. To simplify the interpretation of results across the multiple superfamilies in this study, only enzymes comprised of single domains and that catalyze unimolecular reactions were investigated. Automation of the analysis allows us to describe overall trends in functional conservation and variation across a large number of superfamilies. A descriptive representation of conserved enzyme molecular functions using chemical structures and SMILES strings [30],[31] is also provided. This representation should be useful for annotating new members of superfamilies discovered in sequencing projects and for characterizing new superfamilies.

## Results

Results are presented for 42 superfamilies from the Structural Classification of Proteins (SCOP) database [32]. These superfamilies meet the following criteria: (1) they consist of only single-domain enzymes that (2) perform only unimolecular reactions (or reactions with two substrates, of which one is water), and (3) the superfamilies include at least two different reactions (representing at least two different E.C. numbers) for which substrate and product information are available in the enzyme database BRENDA [33]. Sufficient data were available in BRENDA (the third criterion) for 46.2% of the superfamilies meeting the first two criteria. These 42 superfamilies include representatives of six of the seven SCOP fold classes; the only fold class not represented is the membrane proteins class. The enzymes in these 42 superfamilies represent a substantial proportion of the diversity of enzyme function, covering 25.4% of EC classes defined by the first two digits (subclasses) and 18.7% of EC classes defined by the first three digits (sub-subclasses). Conservation patterns were examined using only substrates and products as the data available in BRENDA were not sufficient to consider other aspects of reaction conservation, such as transition states and intermediates.

**Figure 1:**
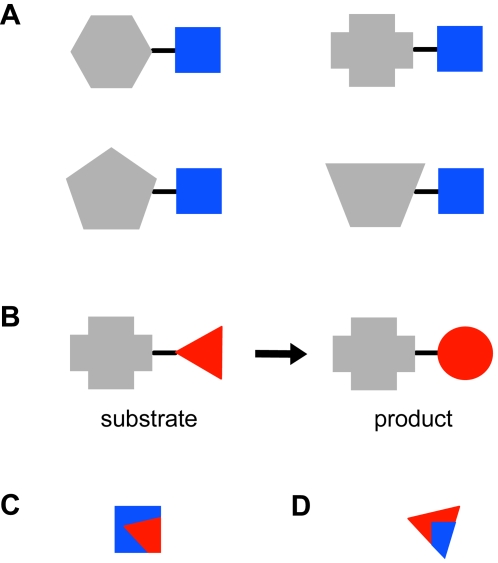
Substructure definitions. (A) The conserved substructure (c) (blue square) is the maximal set of bonds that are present in all the substrates of a superfamily and their adjacent atoms. (B) Reacting substructure (r) (red triangle) is calculated by finding the maximal set of bonds in a substrate that are not present in the product, their adjacent atoms, and the atoms that form new bonds in the product. (C) *f*_c_ is the fraction of the conserved substructure (blue square) that is reacting (red triangle overlap) and is calculated as (r ∩ c)/c. (D) *f*_r_ is the fraction of the reacting substructure (red triangle) that is conserved (blue square overlap) and is calculated as (r ∩ c)/r.

**Figure 2:**
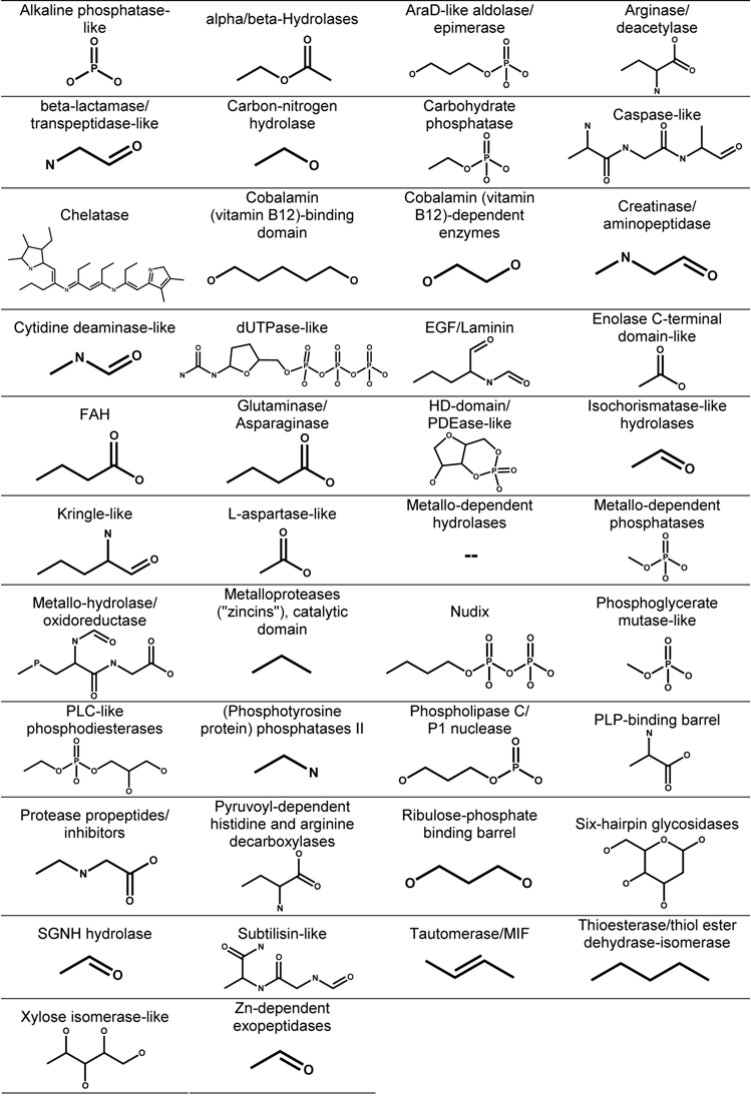
Summary of superfamilies and their conserved substrate substructures. Because the portion of the conserved substructure that is reacting often varies among members within one superfamily, we do not highlight the reacting substructure in this figure. (See Figure 4 for plots of the distribution of this variation over all superfamilies and Table S2 for values of variation for each superfamily.)

Our goal was to determine the molecular features that the substrates of a superfamily share and whether the shared features are involved in the reactions catalyzed by that superfamily. Thus, for each superfamily, we identified the conserved substructure, defined as the set of bonds and their connected atoms that are present in all substrates of the superfamily (Figure 1A). These conserved substructures for the 42 superfamilies in our dataset are shown in Figure 2. Additional information about the diversity and conservation of functions in these superfamilies is provided in a hyperlinked table in the supplementary information online (Table S1). Moreover, for each enzyme's substrate(s), we found the reacting substructure by determining what atoms and bonds change between the substrate and the product (Figure 1B). For each enzyme, we then determined whether the conserved substructure overlaps with the reacting substructure and by how much. This overlap was quantified by calculating the fraction of the conserved substructure that is reacting (*f*_c_) (Figure 1C, Table S2) and the fraction of the reacting substructure that is conserved (*f*_r_) (Figure 1D, Table S2). Results for these measures of overlap are presented with respect to both the number of atoms and the number of bonds.

For a given superfamily, the average *f*_c_ and *f*_r_ calculated using atoms often differ from the values obtained using bonds (Table S2). This difference arises because the number of bonds is frequently not proportional to the number of atoms in molecular structures (e.g., one bond consists of two atoms while three atoms can be connected by three bonds; a cyclic structure will have a different number of bonds compared to non-cyclic structure with the same number of atoms). In addition, different types of reactions vary in the ratio of atoms and bonds that are involved in the reaction (e.g., a lyase may break one bond involving two atoms while an intramolecular transferase may involve one bond and three atoms). Because both are valid measures of substructure size, both are provided in this report.

**Figure 3:**
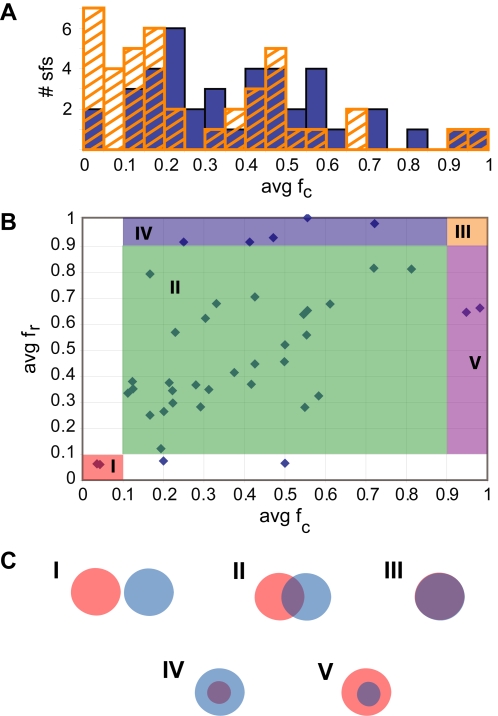
Distribution of overlap between conserved and reacting substructures. (A) Distribution of average fraction of conserved substructure that is reacting. For bonds (orange stripe) and for atoms (blue solid) (B) scatter plot of average *f*_r_ versus *f*_c_. The average *f*_c_ and average f_r_ are calculated using atoms. Each superfamily is represented by a blue diamond. The plot is colored to orient the reader within the plot and to roughly indicate where the different overlap patterns fall. (I) Completely nonoverlapping (red), (II) partially overlapping (green), (III) completely overlapping (orange), (IV) reacting is part of conserved substructure (blue), (V) conserved is part of reacting substructure (purple). (C) Five types of overlap patterns. The conserved substructure (blue circle) can have the following overlap (purple) with the reacting substructure (red circle): (I) completely nonoverlapping, (II) partially overlapping, (III) completely overlapping, (IV) reacting is part of conserved, (V) conserved is part of reacting.

The distribution of average *f*_c_ for the set of superfamilies (Figure 3A) indicates that there is a continuum among the superfamilies in how much of the conserved substructure is reacting, with superfamilies ranging from having little to having most of the conserved substructure participating in the reaction. This trend is observed regardless of whether we use atoms or bonds in our calculations of average *f*_c_. The results also show that all superfamilies with a conserved substructure have an average *f*_c_ above zero, indicating that at least part of the conserved substructure is involved in the reaction.

Only one superfamily in our study set, the superfamily defined by SCOP as the metallo-dependent hydrolase superfamily, also known as the amidohydrolase superfamily [34],[35], has substrates so diverse that they do not share a common substructure of even a single conserved bond. Detailed analysis of the superfamily, including analysis of differences in the overall functions, how active site motifs are used for catalysis, and other factors such as metal ion dependence, suggests that this group may be more properly considered as multiple superfamilies (Brown and Babbitt, in preparation).

Plotting *f*_r_ against *f*_c_ illustrates the distribution of superfamilies (Figure 3B) across different patterns of overlap (Figure 3C) in the reacting and conserved substructures. For simplicity, only the data calculated using atoms is provided in Figure 3B. The values for each superfamily, calculated using both atoms and bonds, are provided in Table S2. The different regions in Figure 3B are intended merely to orient the reader to the range of variation across multiple superfamilies rather than to infer distinct categories implying fundamental differences between the superfamilies in different regions.

**Figure 4:**
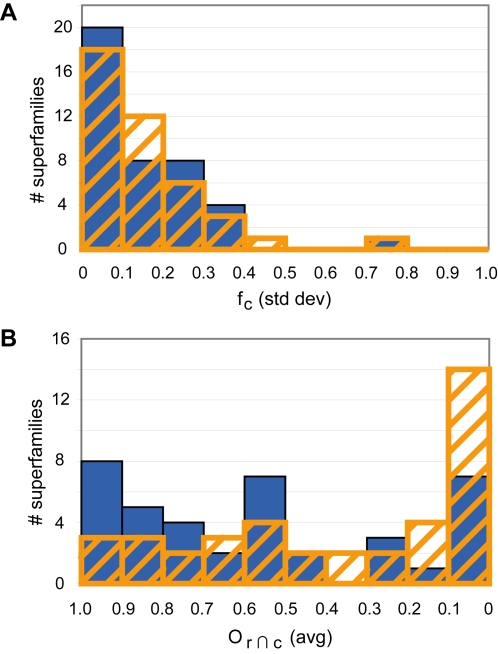
Variation in the overlap between the conserved substructure and reacting substructure. (A) Variation in the fraction of the conserved substructure that is reacting. Distribution of the observed standard deviation in *f*_c_ within each superfamily, for bonds (orange stripe) and atoms (blue solid). (B) Variation in which part of conserved substructure is reacting. Average pairwise overlap in the reacting and conserved substructure (o_r ∩ c_), for bonds (orange stripe) and atoms (blue solid). In both plots, superfamilies with less variation can be found on the left side of the distributions and those with more variation are found on the right.

To determine whether there are differences in how a conserved substructure is used within a single superfamily, the variation of *f*_c_ within each superfamily was also evaluated (Table S2). Most superfamilies have little variation in how much of the conserved substructure is reacting (Figure 4A). However, there are a few superfamilies with substantial variation in *f*_c_. We also evaluated the level of variation in which part of a superfamily's conserved substructure is used among the different reactions by calculating the average overlap in reacting and conserved substructures (o_r ∩ c_) of every pair of substrates in the superfamily. A flatter distribution and more variation was observed among the superfamilies for the average o_r ∩ c_ (Figure 4B) than for the standard deviation of *f*_c_. The superfamilies that rank highest both in variation in *f*_c_ and o_r ∩ c_ include the carbon-nitrogen hydrolase, metalloproteases (“zincins”) (catalytic domain), and the thioesterase/thiol ester dehydrase-isomerase superfamilies. Superfamilies that have low variation in *f*_c_ and o_r ∩ c_ include the HD-domain/PDEase-like, dUTPase-like, and carbohydrate phosphatase superfamilies.

From these examples of superfamilies with high and low variation in *f*_c_ and o_r ∩ c_, we observe that the superfamilies with high variation tend to have smaller conserved substructures while superfamilies with low variation tend to have larger conserved substructures, though the correlation is not perfect. The superfamilies in the low variation group have phosphate groups in the conserved substructure. These tendencies may arise because different superfamilies and different types of reactions have different propensities for variation and conservation through evolution. Alternatively, variation in how different superfamilies are defined in SCOP may lead to some of the variation observed among these superfamilies. We also note that the set of reactions surveyed in this work represents only a subset of enzyme superfamilies, making it difficult to definitively address these hypotheses and questions. More extensive analyses will be required to confirm and further explore these initial observations.

As new superfamily members are characterized, modifications of these substructure conservation patterns may be required. To provide updates of this information, work is underway to incorporate this information into a searchable resource within our Structure-Function Linkage Database (http://sfld.rbvi.ucsf.edu/) [15]. Additional data generated in this study, including reacting substructures and how they overlap with conserved substructures for individual superfamily members, are available from the authors upon request. As described below, our method can also be used to determine conserved functional characteristics for superfamilies that have not yet been characterized. Programs and scripts required to perform these analyses are also available upon request.

## Discussion

Our analysis of the conservation of substrate substructures in enzyme superfamilies precisely determines aspects of chemical transformations that are conserved during divergent evolution. As such, it provides a view of conservation and divergence different from the view afforded by more common types of studies focused on enzyme sequences and structures. While our dataset of superfamilies and their associated substrates, products, and reactions is large, it is still limited as only single domain and unimolecular enzymes and superfamilies with sufficient data available were considered. Nevertheless, the results suggest a continuum in how enzyme superfamilies have evolved, from the reacting substructure being mostly conserved to being only slightly conserved (Figure 3A). Moreover, these superfamilies span a wide range in patterns of overlap (Figure 3B and 3C).

Previously, both large-scale and focused studies of enzyme evolution have recognized two primary models of how function is conserved. In the retro- or substrate-conserved model of enzyme evolution, Horowitz's original hypothesis describes how an existing enzyme in a pathway is duplicated and then evolves to convert new molecules into the substrate for the original enzyme in a metabolic pathway [36],[37]. In the resulting pathway, the newly evolved enzyme will function to provide a reaction required upstream of the original enzyme (i.e., the product of the newly evolved enzyme would be the substrate for the parent). In the second model, chemistry-constrained evolution, the ancestral enzyme, which can be from any pathway, is already promiscuous for or performs a fundamental type of chemistry (often a partial reaction) in common with the function of the daughter enzyme. The aspect of catalysis shared by the ancestral and daughter enzymes is maintained through conservation of structural features such as active site residues [1],[17],[38]. The key difference between these two models is in the pattern of function conservation within each. Related proteins that have diverged via the retro- or substrate-conserved model will bind substrates in common while the chemical reactions with those substrates differ. In the chemistry-constrained model, divergence can give rise to large superfamilies performing many different reactions. Members of such superfamilies will have conserved some aspect of the chemical reaction, which is often a partial reaction, while the substrates they use and their overall chemical reactions differ.

For the most part, the previous studies that have classified superfamilies into one or the other of these categories have been limited either in their scope (see the review by Glasner et al. for examples [39]) or in the type of data used [8],[9],[20],[21]. Although our current work cannot be directly compared with these previous analyses because of differences in methodologies, our results suggest that the evolution of enzyme function is too complex to be described by a few distinct categories. Instead, we see large variations in the patterns of substrate conservation across the set of superfamilies investigated in this study. Also, in these superfamilies, conserved substructures are not entirely reacting nor are they entirely non-reacting. This observation also suggests that the reacting and non-reacting substructures, the latter often including the part of the substrate that has binding interactions with the enzyme, are simultaneously relevant to the evolutionary process and should be analyzed together. Consistent with our observations, a recent network-based analysis of the evolution of metabolism concludes that the two models previously used to describe enzyme evolution are not mutually exclusive or independent [40].

Variations observed within individual superfamilies suggest additional complexity in the evolution of function and how conserved substrate substructures are used in catalysis. Although within most of the superfamilies we studied there is little variation in the extent to which conserved substructures are involved in the reaction (Figure 4), the observation of some variation, and in a few cases, considerable variation, demonstrates that even members of the same superfamily may not proceed with the same pattern of evolution.

As discussed in the sections below, these results also suggest potentially important implications for the analysis of individual superfamilies, functional annotation, and value of evolutionary information in providing guidance for enzyme engineering.

### Functional Annotation of Superfamilies and Enzymes

By automating the analysis of enzyme substrates and reactions, the methodology introduced in this work facilitates the analysis of previously unstudied enzyme superfamilies. This effort contrasts with previous analyses of enzyme superfamilies to determine patterns of functional conservation that have been highly labor-intensive, involving extensive manual analysis of reactions and literature-based curation of functional properties (see the SFLD, http://sfld.rbvi.ucsf.edu/, for examples). The substructures conserved among the substrates of all members of a superfamily (Figure 2) provide annotation information that describes how function has been conserved in each of these superfamilies. The certainty of these superfamily annotations will depend, however, on how well the range of substrates in each superfamily has been sampled. Thorough substrate sampling may be especially critical for complex superfamilies that include many different catalytic functions. While we have used all available reaction information in our analyses, the sampling of superfamily reactions may still be incomplete. As new reactions are discovered through the sequencing of new genomes and metagenomes, these results can be updated and improved.

Despite these limitations, the characterization of superfamily-conserved substructures presented here facilitates the annotation of individual sequences on a large scale, helping to address the need for new strategies for automated function annotation. This issue has become more pressing as the number of sequenced genomes increases and the era of metagenomics moves into high gear [41]. Sequences that can be classified into a superfamily but not into a specific family can be annotated with the substructure common to all characterized members. In these cases, often found in complex superfamilies exhibiting broad diversity in enzyme function, this may be the only level at which accurate annotation can be achieved, as insufficient information may be available to support annotation of a specific reaction or substrate specificity.

**Figure 5:**
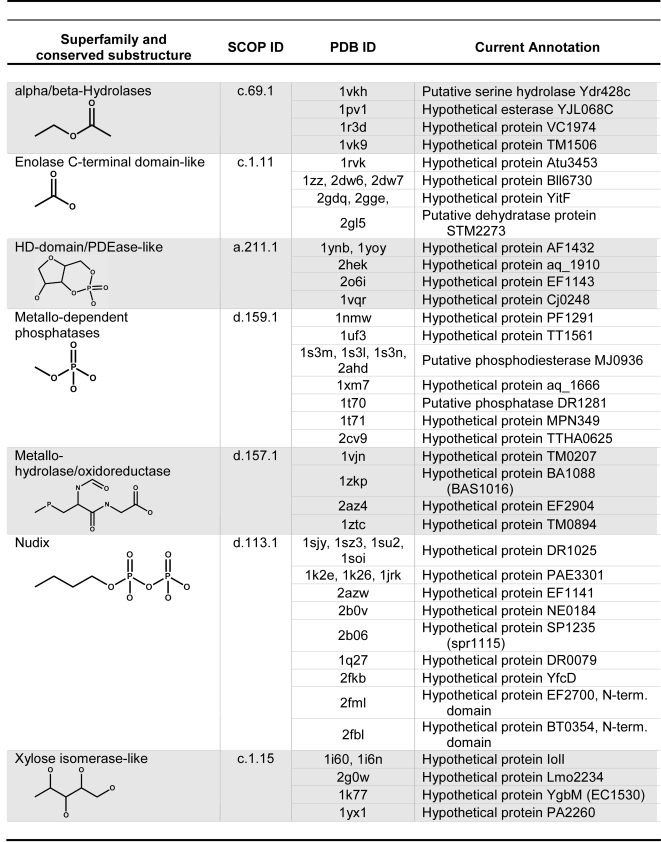
Protein structures with unknown function can be annotated with superfamily-conserved substructures. This partial list includes superfamilies with between four and nine proteins of unknown function. See Table S3 for the full list.

While substructure-based annotation does not by itself suggest a specific enzyme function, this information can be used as a starting point for additional analyses to determine specific function. For example, many structures have been solved through structural genomics efforts, but their functions remain unknown [42]. We have compiled a list of structures that have been classified into the SCOP superfamilies analyzed in this study, but have unknown functions. These structures, many of them from structural genomics projects, can be at least minimally annotated with the substructure identified here as conserved across that superfamily, illustrated by the examples given in Figure 5 (see Table S3 for the complete list). Using this information, characteristics of ligands likely to be bound or turned over by these proteins can be inferred, providing guidance for biochemical studies to determine specificity. These data also provide information about classes of small molecules that may be useful for co-crystallization trials to aid in solving the structures of these proteins or to capture them in functionally relevant conformations.

The variation found within superfamilies presents a caveat to be considered when using these substructures for function annotation. While most of the superfamilies analyzed here have conserved substructures that are used consistently among the different superfamily members (Figure 4), there are a few superfamilies that have significant variation in the degree to which the conserved substructure is used in the reactions. These superfamilies can be expected to be more difficult cases for function prediction since their variability makes it more difficult to determine conserved aspects of function. In contrast, superfamilies with less variation in the degree to which the conserved substructure is used in the reaction are expected to be more straightforward cases for function prediction.

### Guidance for Protein Engineering

**Figure 6:**
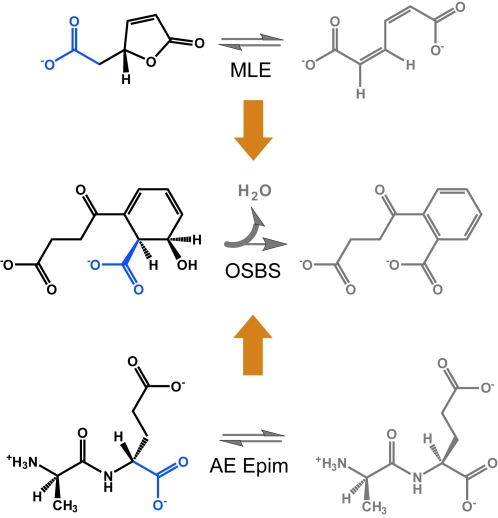
Enzyme engineering strategy. Two previously demonstrated examples using superfamily analysis to guide engineering of enzymes to perform new functions [56]. In the top example, error-prone PCR resulted in a single point mutation of muconate lactonizing II (MLE) enzyme, which enabled it to catalyze the *o*-succinylbenzoate synthase (OSBS) reaction (*k*_cat_/*K*_M_ (M^−1^ s^−1^) = 2×10^3^). In the lower example, a single mutation was rationally designed based on comparison of the active sites of Ala-Glu epimerase (AEE) and *o*-succinyl benzoate synthase (OSBS). The mutant that was generated enabled this enzyme to catalyze the OSBS reaction as well (*k*_cat_/*K*_M_ (M^−1^ s^−1^) = 12.5). In both of these examples, the superfamily conserved substrate substructure (blue) and associated partial reaction were not changed during the engineering experiment. The changes in the reaction that were made are in the portion of the substrates that are not conserved in the superfamily (black). The diverse products of the native MLE, OSBS, and AEE reactions are also shown (grey).

Understanding the patterns of functional conservation associated with the evolution of functionally diverse enzyme superfamilies can provide useful information for guiding enzyme engineering experiments in the laboratory [43]. Using as a starting template for design or engineering an enzyme that already “knows” how to perform a critical partial reaction or how to bind a required substrate substructure ensures that some of the machinery required to perform a desired function is already in place. Although still daunting, the task then simplifies to modifying the enzyme to bind and turn over a new substrate that contains the substructure consistent with the underlying capabilities of the superfamily. As a corollary, aspects of function that have been conserved in all members of a divergent superfamily may be difficult to modify by in vitro engineering [43],[44]. Using such a strategy in a proof-of-concept study, two members of the enolase superfamily were successfully engineered to perform the reaction of a third superfamily member [45]. As shown in Figure 6, the superfamily-conserved substructure and the partial reaction associated with that substructure were not changed in these experiments. Rather, engineering the template proteins to perform the target reaction involved changing each to accommodate binding the part of the substrate that is unique to the new reaction desired.

To allow for generalization of this approach, our analysis provides for all of the superfamilies that we investigated 1) the parts of an enzyme's substrate and reaction that are not conserved among related enzymes, which, provided they can be associated with regions of a target structure that interact with them, may point to structural features amenable to engineering, and 2) the parts of the substrates that are conserved across all members of a superfamily, which may point to regions of the structure that may not be easily changed without loss of function or stability [46].

### Future Directions for Substructure Analysis

In this study, requirements for a sufficiently large sample of enzyme reactions for a comprehensive analysis restricted us to using only substrates and products. However, enzyme substrates can undergo intermediate changes during catalysis that are not adequately captured by looking only at substrates and products. In some reactions, such as those in the enolase superfamily [47], some portions of the substrate change and revert back to their original configuration during the reaction; these types of transformations are undetectable in the study described here. The enolase superfamily represents a well-characterized example of chemistry-conserved evolution. However, because our analysis does not currently detect such substrate changes, the average *f*_c_(atoms) for the enolase superfamily is 0.31 and the average *f*_c_(bonds) for the enolase superfamily is 0.34, which places this superfamily in the middle of the distribution among our superfamilies for these measures of overlap. Being able to detect the full extent to which structures change during a reaction would provide a better picture of substructure conservation in superfamilies like the enolase superfamily. But this will require compilation of additional data to capture all of the partial reactions involved in a given overall reaction, including structures of reaction intermediates. Emerging data resources, such as MACiE [16] and the SFLD [15], currently seek to catalog information about reaction steps and mechanisms. However, because this process is labor-intensive and often hampered by disagreement or ambiguity in the literature regarding the specific mechanisms of some reactions, these data resources are not yet sufficiently populated to support such broader analyses. As these types of resources grow, we are optimistic that the information required to analyze reaction mechanisms more fully will become increasingly available. Although it is beyond the scope of this study, correlating the conservation patterns we see in enzyme substrates with the conservation patterns in the sequence and structures of the enzymes themselves would also be a valuable extension for these analyses.

Finally, recent progress has been made in using in silico docking of small molecules to enzyme structures to infer molecular function. In one such study, a library of high-energy reaction intermediates was generated and used to predict substrate specificity of enzymes in the amidohydrolase superfamily [48]. As these methodologies are further developed, incorporation of predicted reaction intermediates into substructure analysis could improve prediction of substructures that are reacting. In addition to benefiting from such recent advances in docking, the type of analysis presented here may in turn be used to improve applications of docking to predicting substrate specificity in enzymes. Several such studies have recently focused on predicting functional specificity in the enolase [49],[50] and amidohydrolase [51] superfamilies using knowledge about conserved substrate substructures from earlier analyses [15],[52] to construct focused ligand libraries for docking. We expect that the set of conserved substructures generated by our analysis can be used similarly to guide the construction of chemical libraries of ligands to improve prediction of substrate specificity in other superfamilies.

### Conclusions

This study presents an automated method for analysis of superfamilies to determine the conserved aspects of their functions, represented by patterns of substrate conservation. Our results show that superfamilies do not fall into discrete and easily separable categories describing how their functions may have evolved. Rather, the conserved substructures determined in this analysis define superfamily-specific conservation patterns. These results enable precise prediction of functional characteristics at the superfamily level for complex superfamilies whose members perform many different but related reactions, even when the evidence is insufficient to support more specific annotations of overall reaction and substrate specificity. For applications in enzyme engineering, we expect that the identification of the aspects of function that have been most and least conserved during natural evolution will provide guidance for identifying the structural elements of a target scaffold that are most and least amenable to modification, thereby informing engineering strategies for improved success.

## Methods

### Dataset—Enzyme Superfamilies

For our analyses, we used a subset of superfamilies from SCOP, a database of manually classified protein superfamilies, filtered based on criteria chosen to be most informative about enzyme evolution at high levels of functional divergence. We included only superfamilies of single-domain enzymes with significant functional information in SCOPEC, a subset of SCOP with verified EC numbers, and in BRENDA, the most comprehensive database of enzyme experimental results. Although many enzymes and proteins function as multi-domain units, the nature and organization of which can affect the specificity and regulation of enzymes [53], for this study, we chose to use only single-domain enzymes as this allowed us to clearly assign a single function to one domain. We included examples of enzymes known to have multiple structural domains only when the composite acts as a single functional unit (e.g., the enolase superfamily).

To ensure that the members of each superfamily were sufficiently divergent in function to analyze conservation of their substructures, only superfamilies annotated with at least two different EC numbers were investigated. Compared to unimolecular reactions, bimolecular reactions have considerably more complex chemical and kinetic mechanisms for how substrates interact with the enzyme's catalytic site (i.e., in what order different substrates bind). Because these variations would have greatly complicated the analysis, we excluded superfamilies with any reactions that were not unimolecular. Using the top level of the EC annotation, superfamilies were selected in which all the characterized members belong to any one of the following classes: hydrolases (EC numbers 3.x.x.x), lyases (EC numbers 4.x.x.x), and isomerases (EC numbers 5.x.x.x).

Experimentally verified substrate and product data were taken from the licensed version of the BRENDA database (release 6.2) [54]. Reactions were excluded in which (1) the product(s) had more than five (non-hydrogen) atoms more than the substrate or (2) substrates and products both had three or fewer (non-hydrogen) atoms. Reactions in the first category are likely to be erroneous because they are not properly balanced. Reactions in the second category are unlikely to be informative for the analysis because they contain so few atoms.

### Definitions

A “conserved substructure” (Figure 1A) contains the maximal sets of bonds in a substrate that are present in all the substrates of a superfamily, plus their adjacent atoms. In all our analyses, we considered only bonds consisting of two atoms, neither of which is a hydrogen. The “unconserved substructure” is the set of bonds in a substrate that are not in the conserved substructure, plus their adjacent atoms. An atom can be in both the conserved and unconserved substructure if it is adjacent to both a bond in the conserved substructure and a bond in the unconserved substructure.

A “reacting substructure” (Figure 1B) consists of the bonds in a substrate that are not present in the product, their adjacent atoms, and any atoms that become connected in new bonds in the product. In the case of a racemization reaction, in which the chirality of an atom center changes, the reacting substructure is defined as including the chiral atom that changes in the reaction, the four adjacent bonds and their adjacent atoms. The “nonreacting substructure” is the set of bonds in a substrate that are also present in the product and their adjacent atoms. An atom can be in both the reacting and nonreacting substructure if it is adjacent to both a bond in the reacting substructure and a bond in the nonreacting substructure.

### Finding the Conserved Substrate Substructure

The substrate substructure conserved among all characterized members of each superfamily was calculated using the maximal common substructure (MCS) algorithm implemented in the Chemistry Development Kit (CDK) [55], an open source Java toolkit for manipulating small molecules. The molecules are represented as graphs in which the nodes represent atoms and the edges represent bonds. Each node is labeled with an atom type and each edge is labeled with the two atom types of the connected atoms and the bond order. This algorithm finds, for a pair of molecules, the maximum common substructure (MCS) present in both molecules. We extended this to find the MCS for the set of all known substrates for a superfamily. In this initial analysis, we treated different atoms as dissimilar as long as the element type was different and bonds as different when the bond order and the two pairs of connected atoms were not identical. The only exception to this rule was made for phosphate and sulfate groups, which we treated as similar in the substrate conservation analyses. Our code allowed for the possibility of multiple unconnected MCSs by representing them as an unconnected graph with each connected portion corresponding to one MCS. Although some of the pairwise MCSs contain multiple unconnected subgraphs, none of the superfamily-conserved substructures contain such multiple unconnected MCSs. Finally, each substrate has a unique unconserved substructure defined as the set of edges not present in the conserved substructure and the atoms adjacent to these edges.

### Finding the Reacting Substrate Substructure

For each enzymatic reaction in which both the substrate and its corresponding product(s) are known, we calculated the non-reacting substructure by finding the MCS between the substrate and the product(s). The reacting substructure is the set of edges in the substrate that are not present in the product, plus the atoms adjacent to these edges. The reacting substructure also includes atoms that form new bonds in the product.

### Overlap between Reacting and Conserved Substructures

To quantify the overlap between the reacting and conserved substructures, for each reaction in our dataset, we calculate *f*_c_ (Figure 1C), the fraction of the conserved substructure that is reacting and *f*_r_ (Figure 1D), the fraction of the reacting substructure that is conserved. The values for *f*_c_ and *f*_r_ are calculated in two ways, using atoms or bonds, and the results for both are reported as they provide different but useful views of the data. *f*_c_ for bonds is determined by dividing the number of bonds that are in both the conserved and the reacting substructures (r ∩ c) by the number of bonds in only the conserved substructure. *f*_c_ for atoms is determined similarly, using the number of atoms instead of bonds. Likewise, *f*_r_ for bonds is determined by dividing the number of bonds that are in both the conserved and the reacting substructures by the number of bonds in only the reacting substructure; this value was also calculated using atoms. For each enzyme in the BRENDA database, there may be multiple substrates with corresponding reactions that have been characterized. For these cases, the values of *f*_c_ and *f*_r_ were obtained by averaging all the substrates of each enzyme and then these values were averaged for all the enzymes in each superfamily. We also determined the standard deviation in *f*_c_ and *f*_r_ for the enzymes of each superfamily.

### Variation in Which Substructure Is Reacting

To determine whether the same part of the superfamily-conserved substructure was used in the different reactions of the superfamily, every pair of reactions was analyzed in each of the superfamilies in our dataset. Each reaction has a substrate substructure that is both conserved and reacting (r ∩ c). For each pair of reactions, we calculated how much overlap is observed among the two (r ∩ c) substructures and normalized each of these overlaps by the smallest (r ∩ c) of each pair. The resulting measure of overlap (o_r ∩ c_) was then averaged over every pair of reactions in each superfamily.

## Supporting Information

Table S1 Conserved EC positions and conserved substructures associated with each superfamily. The superfamilies in this table are sorted by [average *fc*(atoms) plus *fc*(bonds)] (as given in Table S2).
(2.53 MB DOC)

Table S2 Overlap between reacting and conserved substructures (*fc* and *fr*). The superfamilies in this table are sorted by [average *fc*(atoms) plus *fc*(bonds)]. *The metallo-dependent hydrolases superfamily does not have a substrate substructure that is conserved in all members of the superfamily. Thus, for this superfamily, *fc*, the fraction of the conserved substructure that is reacting, cannot be calculated.
(0.16 MB DOC)

Table S3 Superfamilies annotation list. These structures are mostly from structural genomics projects. Annotation of these superfamilies with their conserved substructures may provide useful guidance for analyses to determine the function of these proteins or to identify characteristics of ligands useful for co-crystallization attempts.
(0.14 MB DOC)
