# Supplementary material for: Evolutionarily Conserved Substrate Substructures for Automated Annotation of Enzyme Superfamilies
Source: PLoS Comput Biol. 2008 Aug 1;4(8):e1000142. doi: 10.1371/journal.pcbi.1000142 (PMC2453236; doi:10.1371/journal.pcbi.1000142)
Supplement: Table S1 — Conserved EC positions and conserved substructures associated with each superfamily. The superfamilies in this table are sorted by [average fc(atoms) plus fc(bonds)] (as given in Table S2). (2.53 MB DOC) [file pcbi.1000142.s001.doc]

|  |  |  |  |  | | |  |  | | |
| --- | --- | --- | --- | --- | --- | --- | --- | --- | --- | --- |
|  |  |  |  |  | | |  |  | | |
| **Superfamily** |  | **SCOP ID** |  | **Number of** | | |  | **Conserved** | | |
|  |  |  |  | **Reactions** |  | **Unique EC numbers** |  | **EC positions** |  | **Substructure** |
|  |  |  |  |  |  |  |  |  |  |  |
| [Alkaline phosphatase-like](http://scop.mrc-lmb.cam.ac.uk/scop/data/scop.b.d.bad.b.html) |  | c.76.1 |  | 67 |  | 5 |  | [3.1.x.x](http://www.chem.qmul.ac.uk/iubmb/enzyme/EC3/1/) |  | 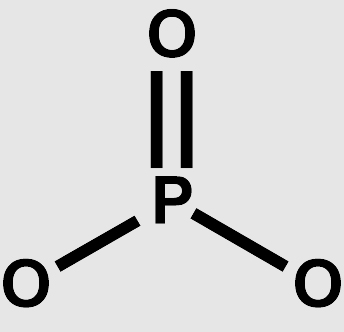 OP(=O)O |
| [SGNH hydrolase](http://scop.mrc-lmb.cam.ac.uk/scop/data/scop.b.d.cg.i.html) |  | c.23.10 |  | 19 |  | 2 |  | [3.1.1.x](http://www.chem.qmul.ac.uk/iubmb/enzyme/EC3/1/1/) |  | 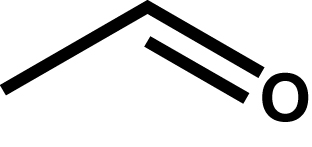 CC=O |
| [Metallo-dependent phosphatases](http://scop.mrc-lmb.cam.ac.uk/scop/data/scop.b.e.cib.b.html) |  | d.159.1 |  | 30 |  | 2 |  | [3.1.3.x](http://www.chem.qmul.ac.uk/iubmb/enzyme/EC3/1/3/) |  | 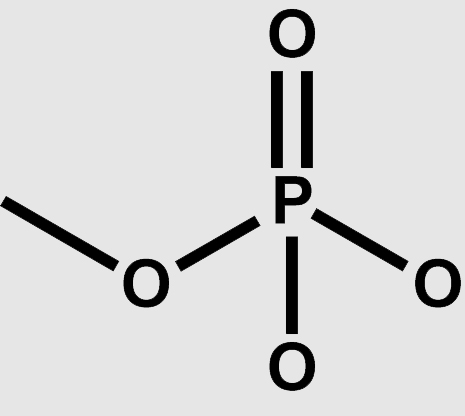 COP(O)(=O)O |
| [Carbohydrate phosphatase](http://scop.mrc-lmb.cam.ac.uk/scop/data/scop.b.f.j.b.html) |  | e.7.1 |  | 17 |  | 4 |  | [3.1.3.x](http://www.chem.qmul.ac.uk/iubmb/enzyme/EC3/1/3/) |  | 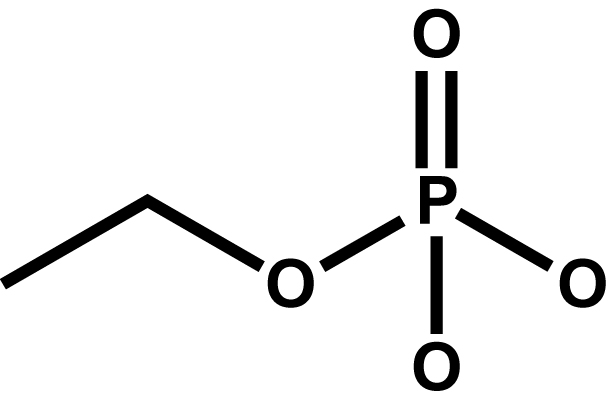 CCOP(=O)(O)O |
| [Cobalamin (vitamin B12)-dependent enzymes](http://scop.mrc-lmb.cam.ac.uk/scop/data/scop.b.d.b.ca.html) |  | c.1.19 |  | 6 |  | 2 |  | [4.2.1.x](http://www.chem.qmul.ac.uk/iubmb/enzyme/EC4/2/1/) |  | 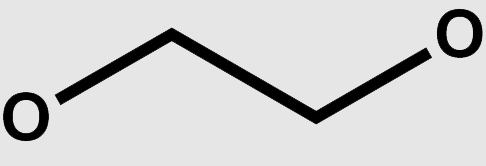 OCCO |
| [Phosphoglycerate mutase-like](http://scop.mrc-lmb.cam.ac.uk/scop/data/scop.b.d.ic.b.html) |  | c.60.1 |  | 58 |  | 5 |  | [x.x.x.x](http://www.chem.qmul.ac.uk/iubmb/enzyme/) |  | 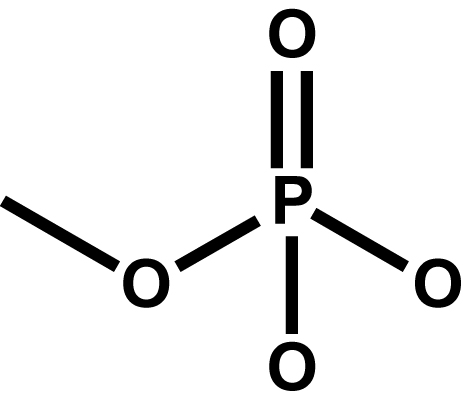 COP(O)(O)=O |
| [Six-hairpin glycosidases](http://scop.mrc-lmb.cam.ac.uk/scop/data/scop.b.b.bib.b.html) |  | a.102.1 |  | 6 |  | 3 |  | [x.x.x.x](http://www.chem.qmul.ac.uk/iubmb/enzyme/) |  | 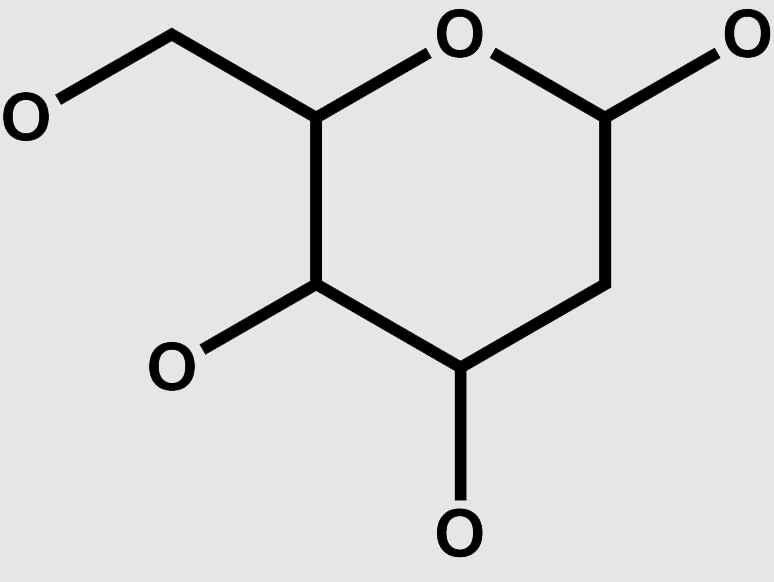 OCC1OC(O)CC(O)C1(O) |
| [alpha/beta-Hydrolases](http://scop.mrc-lmb.cam.ac.uk/scop/data/scop.b.d.jg.b.html) |  | c.69.1 |  | 13 |  | 3 |  | [3.1.1.x](http://www.chem.qmul.ac.uk/iubmb/enzyme/EC3/1/1/) |  | 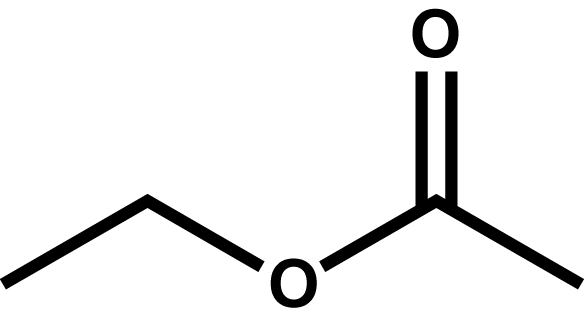 CCOC(C)=O |
| [PLP-binding barrel](http://scop.mrc-lmb.cam.ac.uk/scop/data/scop.b.d.b.h.html) |  | c.1.6 |  | 5 |  | 3 |  | [x.x.x.x](http://www.chem.qmul.ac.uk/iubmb/enzyme/) |  | 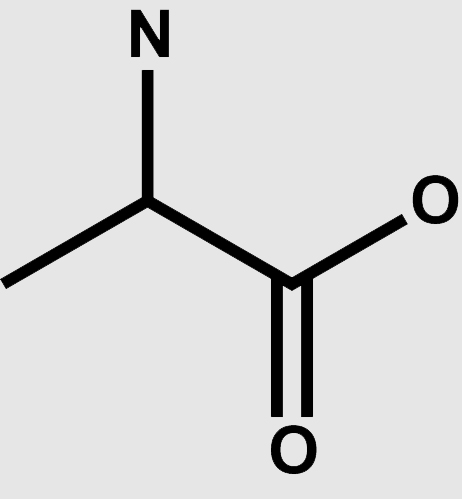 CC(N)C(=O)O |
| [Carbon-nitrogen hydrolase](http://scop.mrc-lmb.cam.ac.uk/scop/data/scop.b.e.cic.b.html) |  | d.160.1 |  | 13 |  | 2 |  | [3.x.x.x](http://www.chem.qmul.ac.uk/iubmb/enzyme/EC3/) |  | 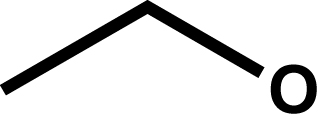 CCO |
| [Creatinase/aminopeptidase](http://scop.mrc-lmb.cam.ac.uk/scop/data/scop.b.e.cde.b.A.html) |  | d.127.1 |  | 31 |  | 4 |  | [3.x.x.x](http://www.chem.qmul.ac.uk/iubmb/enzyme/EC3/) |  | 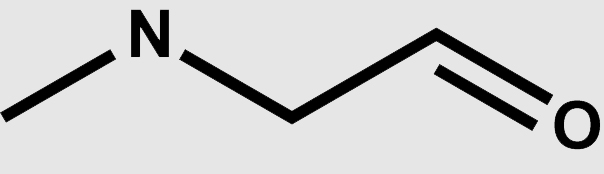 CNCC=O |
| [Metalloproteases ("zincins"), catalytic domain](http://scop.mrc-lmb.cam.ac.uk/scop/data/scop.b.e.big.b.html) |  | d.92.1 |  | 104 |  | 17 |  | [3.x.x.x](http://www.chem.qmul.ac.uk/iubmb/enzyme/EC3/) |  | 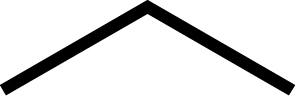 CCC |
| [Nudix](http://scop.mrc-lmb.cam.ac.uk/scop/data/scop.b.e.cbf.b.html) |  | d.113.1 |  | 32 |  | 4 |  | [x.x.x.x](http://www.chem.qmul.ac.uk/iubmb/enzyme/) |  | 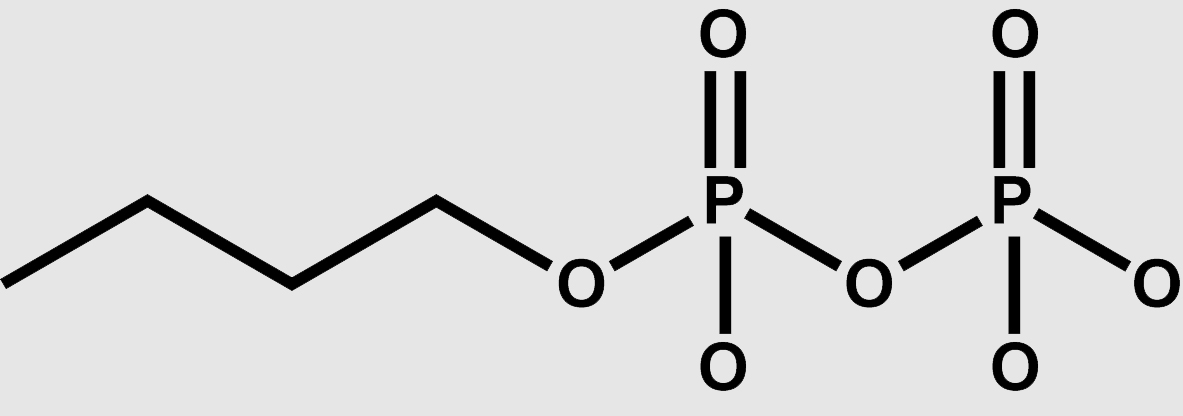 CCCCOP(=O)([O-])OP(=O)(O)O |
| [Phospholipase C/P1 nuclease](http://scop.mrc-lmb.cam.ac.uk/scop/data/scop.b.b.cbf.b.html) |  | a.124.1 |  | 18 |  | 2 |  | [3.1.x.x](http://www.chem.qmul.ac.uk/iubmb/enzyme/EC3/1/) |  | 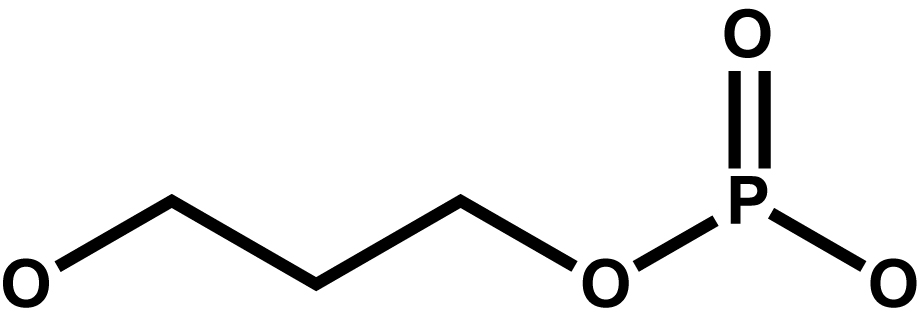 OCCCOP(=O)O |
| [Pyruvoyl-dependent histidine and arginine decarboxylases](http://scop.mrc-lmb.cam.ac.uk/scop/data/scop.b.e.chh.b.html) |  | d.155.1 |  | 6 |  | 2 |  | [4.1.1.x](http://www.chem.qmul.ac.uk/iubmb/enzyme/EC4/1/1/) |  | 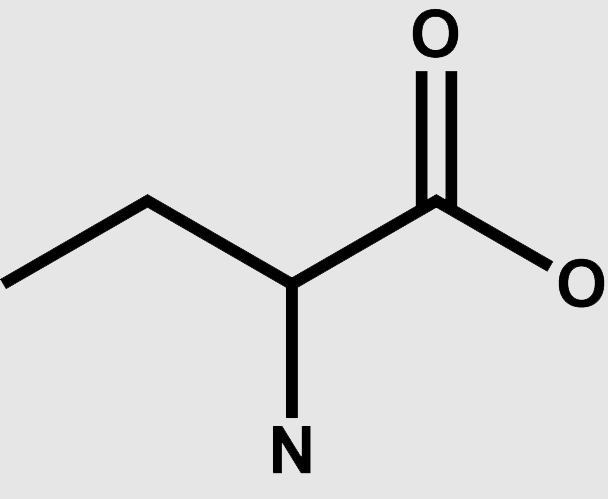 CCC(N)C(=O)O |
| [PLC-like phosphodiesterases](http://scop.mrc-lmb.cam.ac.uk/scop/data/scop.b.d.b.bj.html) |  | c.1.18 |  | 11 |  | 3 |  | [x.x.x.x](http://www.chem.qmul.ac.uk/iubmb/enzyme/) |  | 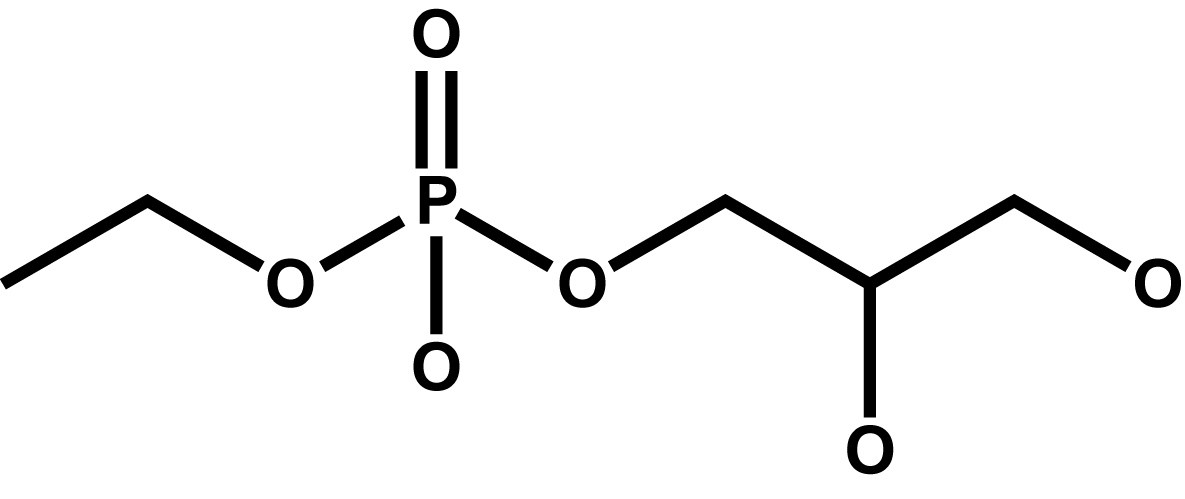 CCOP(=O)(O)OCC(O)CO |
| [dUTPase-like](http://scop.mrc-lmb.cam.ac.uk/scop/data/scop.b.c.bdf.g.A.html) |  | b.85.4 |  | 8 |  | 2 |  | [3.x.x.x](http://www.chem.qmul.ac.uk/iubmb/enzyme/EC3/) |  | 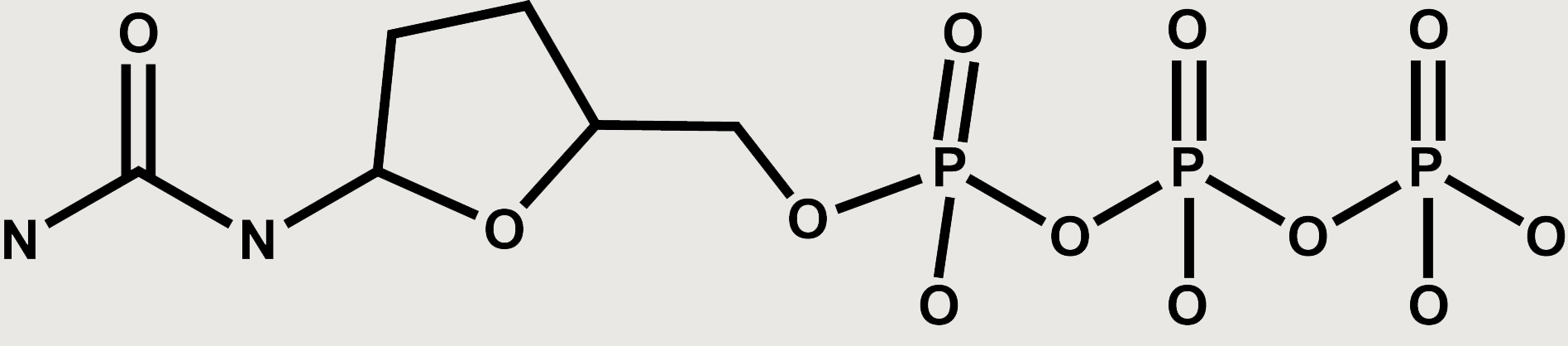 NC(=O)NC1CCC(COP(=O)(O) OP(=O)(O)OP(O)(=O)O)O1 |
| [Tautomerase/MIF](http://scop.mrc-lmb.cam.ac.uk/scop/data/scop.b.e.bgd.b.html) |  | d.80.1 |  | 4 |  | 3 |  | [x.x.x.x](http://www.chem.qmul.ac.uk/iubmb/enzyme/) |  | 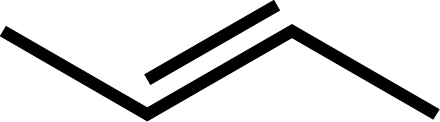 CC=CC |
| [Xylose isomerase-like](http://scop.mrc-lmb.cam.ac.uk/scop/data/scop.b.d.b.bg.html) |  | c.1.15 |  | 5 |  | 3 |  | [x.x.x.x](http://www.chem.qmul.ac.uk/iubmb/enzyme/) |  | 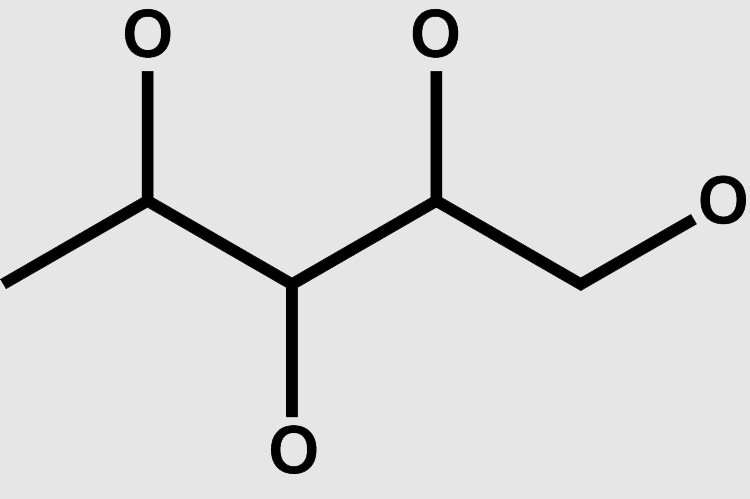 CC(O)C(O)C(O)CO |
| [Zn-dependent exopeptidases](http://scop.mrc-lmb.cam.ac.uk/scop/data/scop.b.d.hg.f.html) |  | c.56.5 |  | 81 |  | 8 |  | [3.x.x.x](http://www.chem.qmul.ac.uk/iubmb/enzyme/EC3/) |  | 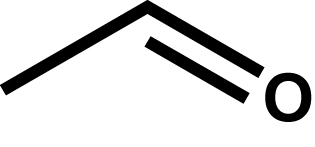 CC=O |
| [Chelatase](http://scop.mrc-lmb.cam.ac.uk/scop/data/scop.b.d.bdb.b.html) |  | c.92.1 |  | 3 |  | 2 |  | [4.99.1.x](http://www.chem.qmul.ac.uk/iubmb/enzyme/EC4/99/1/) |  | 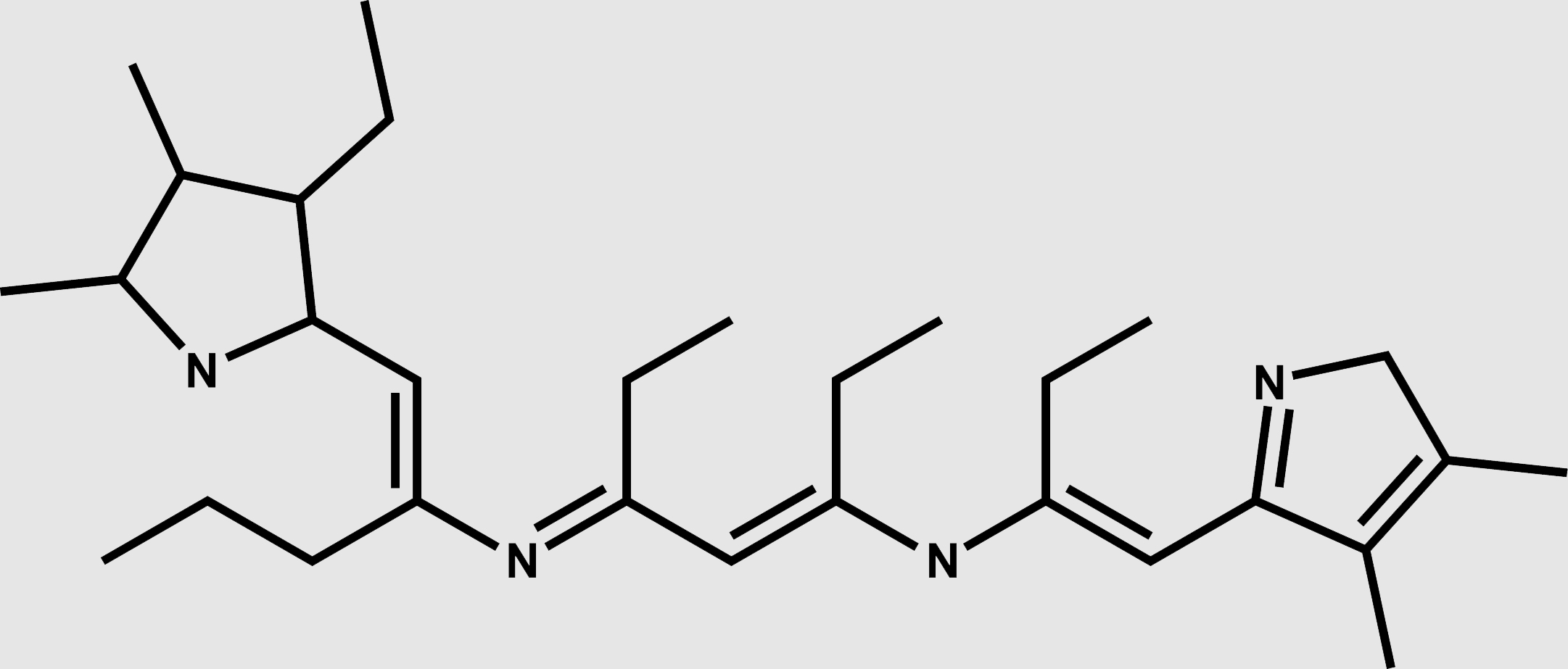 CCCC(=Cc1nc(C)c(C)c1(CC))N=C(CC)C=C(CC) NC(CC)=CC2=NCC(C)=C2(C) |
| [L-aspartase-like](http://scop.mrc-lmb.cam.ac.uk/scop/data/scop.b.b.ccj.b.html) |  | a.127.1 |  | 14 |  | 6 |  | [x.x.x.x](http://www.chem.qmul.ac.uk/iubmb/enzyme/) |  | 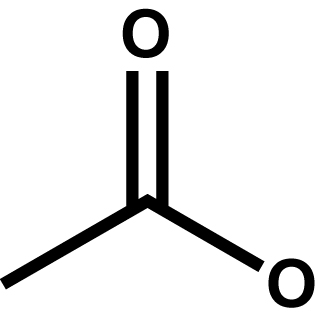 CC(O)=O |
| [Protease propeptides/inhibitors](http://scop.mrc-lmb.cam.ac.uk/scop/data/scop.b.e.bbg.d.html) |  | d.58.3 |  | 21 |  | 3 |  | [3.4.x.x](http://www.chem.qmul.ac.uk/iubmb/enzyme/EC3/4/) |  | 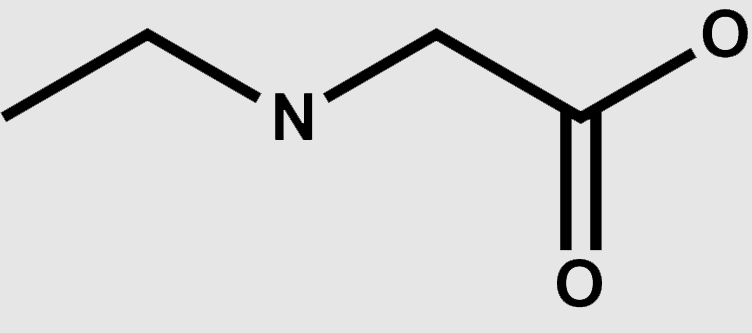 CCNCC(=O)O |
| [Ribulose-phosphate binding barrel](http://scop.mrc-lmb.cam.ac.uk/scop/data/scop.b.d.b.c.html) |  | c.1.2 |  | 10 |  | 5 |  | [x.x.x.x](http://www.chem.qmul.ac.uk/iubmb/enzyme/) |  | 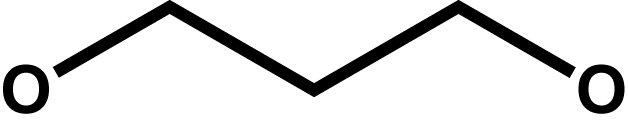 OCCCO |
| [Metallo-hydrolase/oxidoreductase](http://scop.mrc-lmb.cam.ac.uk/scop/data/scop.b.e.chj.b.html) |  | d.157.1 |  | 11 |  | 2 |  | [3.x.x.x](http://www.chem.qmul.ac.uk/iubmb/enzyme/EC3/) |  | 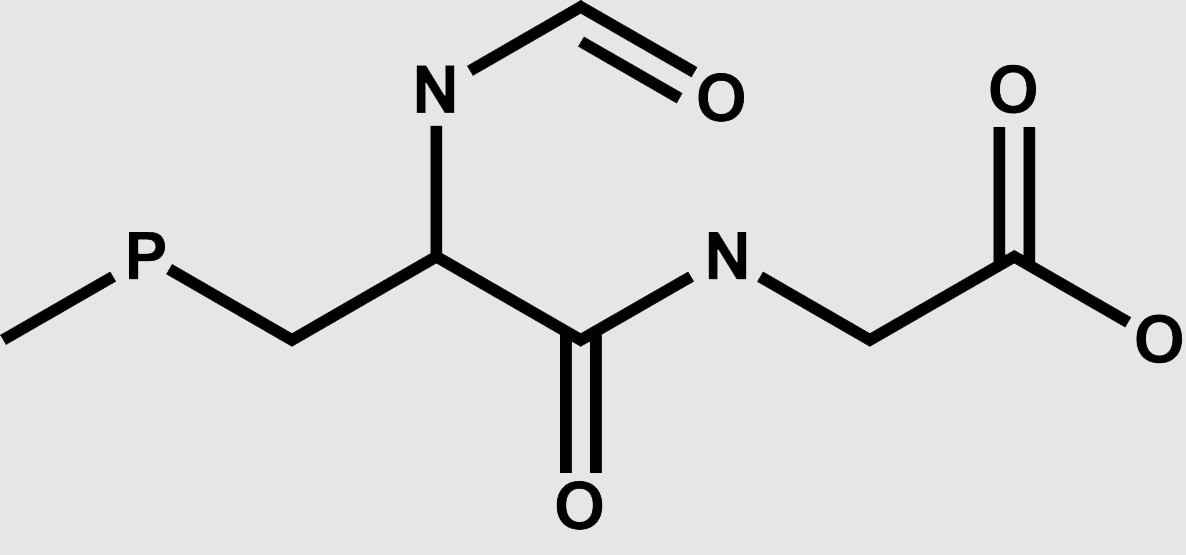 CPCC(NC=O)C(=O)NCC(=O)O |
| [Enolase C-terminal domain-like](http://scop.mrc-lmb.cam.ac.uk/scop/data/scop.b.d.b.bc.html) |  | c.1.11 |  | 26 |  | 6 |  | [x.x.x.x](http://www.chem.qmul.ac.uk/iubmb/enzyme/) |  | 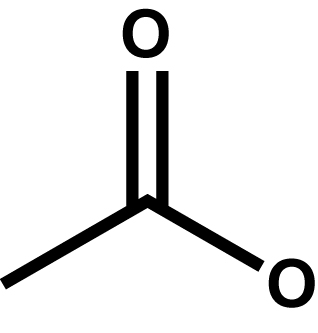 CC(=O)[O-] |
| [Thioesterase/thiol ester dehydrase-isomerase](http://scop.mrc-lmb.cam.ac.uk/scop/data/scop.b.e.ie.b.html) |  | d.38.1 |  | 15 |  | 3 |  | [x.x.x.x](http://www.chem.qmul.ac.uk/iubmb/enzyme/) |  | 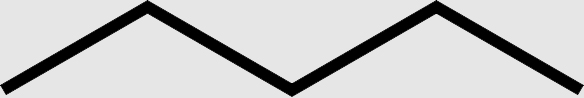 CCCCC |
| [Cobalamin (vitamin B12)-binding domain](http://scop.mrc-lmb.cam.ac.uk/scop/data/scop.b.d.cg.g.A.html) |  | c.23.6 |  | 4 |  | 2 |  | [5.4.99.x](http://www.chem.qmul.ac.uk/iubmb/enzyme/EC5/4/99/) |  | 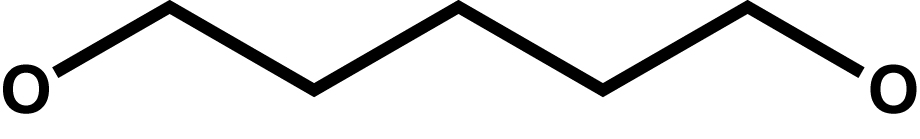 [O-]CCCCC[O-] |
| [Subtilisin-like](http://scop.mrc-lmb.cam.ac.uk/scop/data/scop.b.d.fg.b.html) |  | c.41.1 |  | 6 |  | 2 |  | [3.4.21.x](http://www.chem.qmul.ac.uk/iubmb/enzyme/EC3/4/21/) |  | 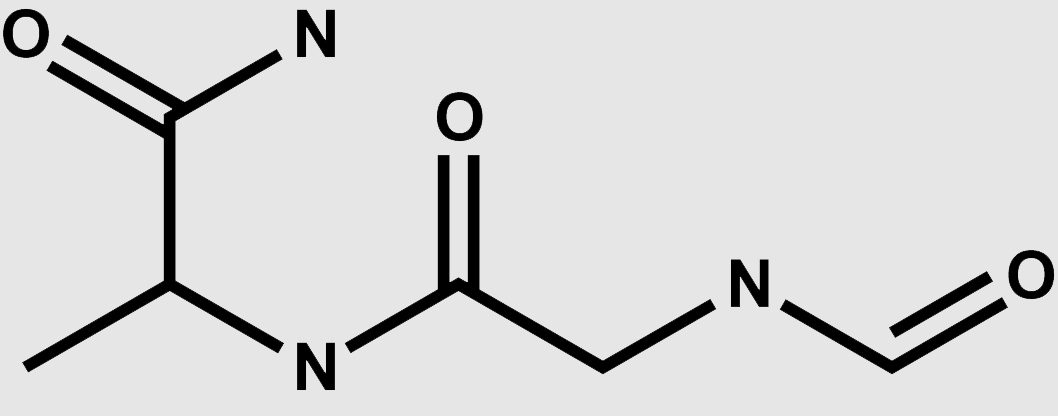 CC(NC(=O)CNC=O)C(N)=O |
| [Kringle-like](http://scop.mrc-lmb.cam.ac.uk/scop/data/scop.b.h.be.b.html) |  | g.14.1 |  | 11 |  | 4 |  | [3.4.x.x](http://www.chem.qmul.ac.uk/iubmb/enzyme/EC3/4/) |  | 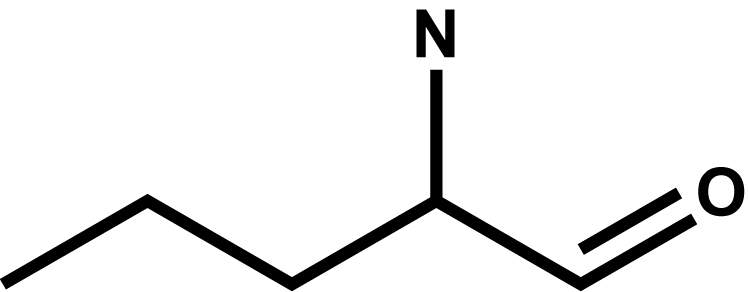 CCCC(N)C=O |
| [beta-lactamase/transpeptidase-like](http://scop.mrc-lmb.cam.ac.uk/scop/data/scop.b.f.dd.b.html) |  | e.3.1 |  | 27 |  | 3 |  | [3.x.x.x](http://www.chem.qmul.ac.uk/iubmb/enzyme/EC3/) |  | 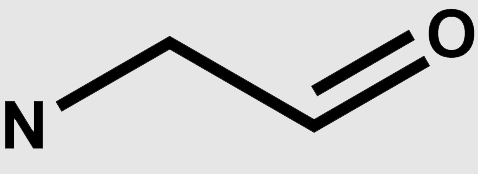 NCC=O |
| [(Phosphotyrosine protein) phosphatases II](http://scop.mrc-lmb.cam.ac.uk/scop/data/scop.b.d.ge.b.html) |  | c.45.1 |  | 9 |  | 2 |  | [3.1.3.x](http://www.chem.qmul.ac.uk/iubmb/enzyme/EC3/1/3/) |  | 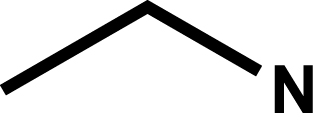 CCN |
| [FAH](http://scop.mrc-lmb.cam.ac.uk/scop/data/scop.b.e.dba.b.A.html) |  | d.177.1 |  | 3 |  | 3 |  | [x.x.x.x](http://www.chem.qmul.ac.uk/iubmb/enzyme/) |  | 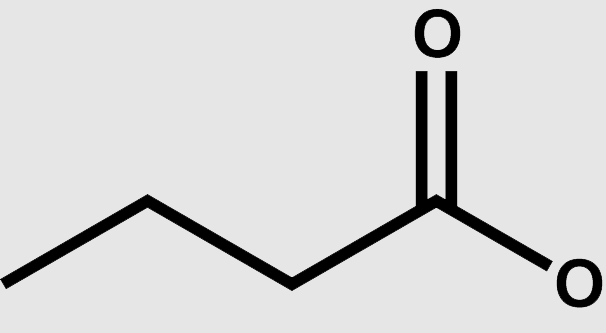 CCCC(O)=O |
| [HD-domain/PDEase-like](http://scop.mrc-lmb.cam.ac.uk/scop/data/scop.b.b.bha.b.html) |  | a.211.1 |  | 10 |  | 2 |  | [3.1.4.x](http://www.chem.qmul.ac.uk/iubmb/enzyme/EC3/1/4/) |  | 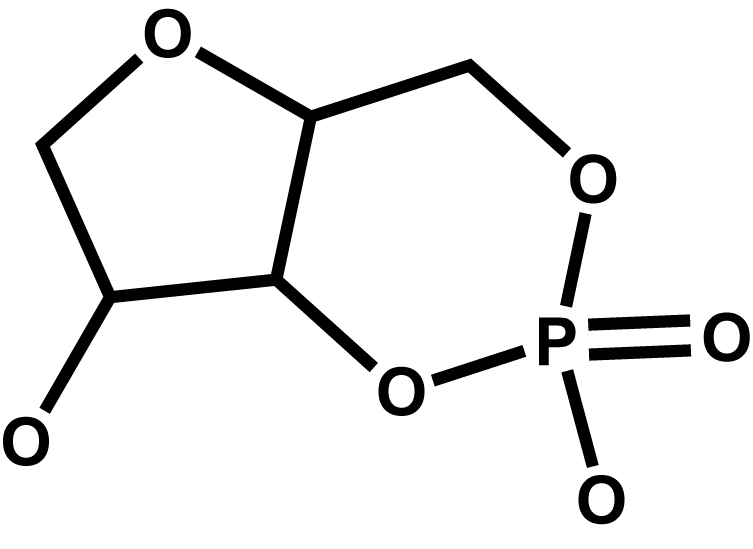 OC1COC2COP(=O)(O)OC12 |
| [Cytidine deaminase-like](http://scop.mrc-lmb.cam.ac.uk/scop/data/scop.b.d.beb.b.html) |  | c.97.1 |  | 26 |  | 3 |  | [3.5.4.x](http://www.chem.qmul.ac.uk/iubmb/enzyme/EC3/5/4/) |  | 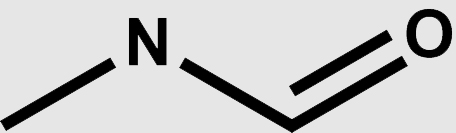 CNC=O |
| [Isochorismatase-like hydrolases](http://scop.mrc-lmb.cam.ac.uk/scop/data/scop.b.d.dj.b.A.html) |  | c.33.1 |  | 2 |  | 2 |  | [3.5.1.x](http://www.chem.qmul.ac.uk/iubmb/enzyme/EC3/5/1/) |  | 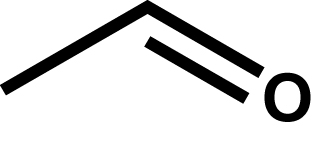 CC=O |
| [Glutaminase/Asparaginase](http://scop.mrc-lmb.cam.ac.uk/scop/data/scop.b.d.bcc.b.A.html) |  | c.88.1 |  | 11 |  | 2 |  | [3.5.1.x](http://www.chem.qmul.ac.uk/iubmb/enzyme/EC3/5/1/) |  | 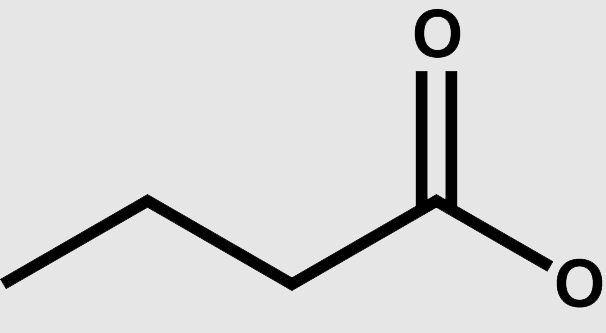 CCCC(=O)O |
| [Caspase-like](http://scop.mrc-lmb.cam.ac.uk/scop/data/scop.b.d.hj.b.html) |  | c.17.1 |  | 13 |  | 2 |  | [3.4.22.x](http://www.chem.qmul.ac.uk/iubmb/enzyme/EC3/4/22/) |  | 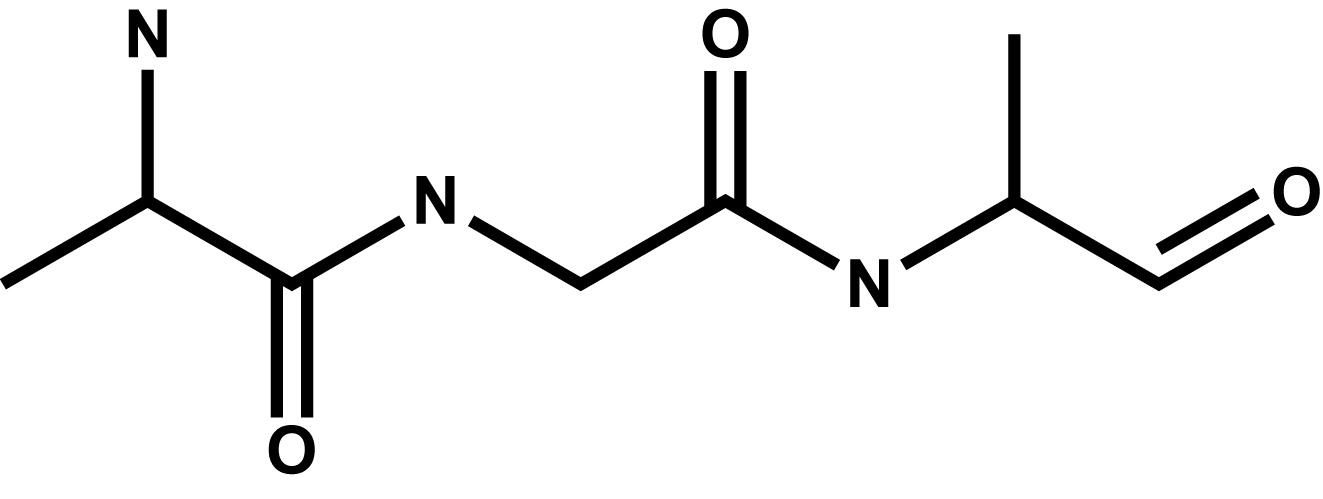 CC(N)C(=O)NCC(=O)NC(C)C=O |
| [AraD-like aldolase/epimerase](http://scop.mrc-lmb.cam.ac.uk/scop/data/scop.b.d.bag.b.A.html) |  | c.74.1 |  | 8 |  | 3 |  | [x.x.x.x](http://www.chem.qmul.ac.uk/iubmb/enzyme/) |  | 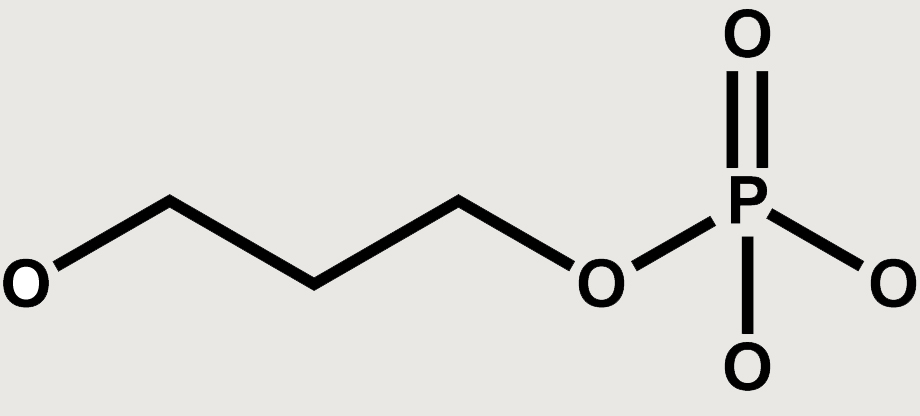 OCCCOP(=O)(O)O |
| [EGF/Laminin](http://scop.mrc-lmb.cam.ac.uk/scop/data/scop.b.h.c.bb.html) |  | g.3.11 |  | 6 |  | 3 |  | [3.4.21.x](http://www.chem.qmul.ac.uk/iubmb/enzyme/EC3/4/21/) |  | 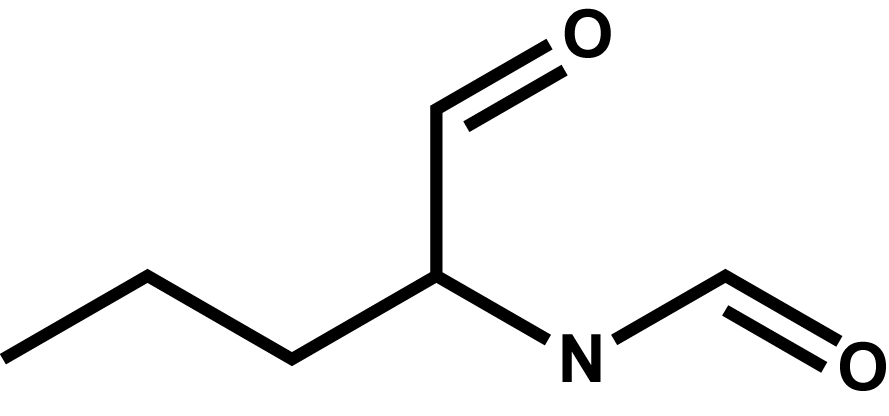 CCCC(C=O)NC=O |
| [Arginase/deacetylase](http://scop.mrc-lmb.cam.ac.uk/scop/data/scop.b.d.fi.b.html) |  | c.42.1 |  | 4 |  | 2 |  | [3.5.3.x](http://www.chem.qmul.ac.uk/iubmb/enzyme/EC3/5/3/) |  | 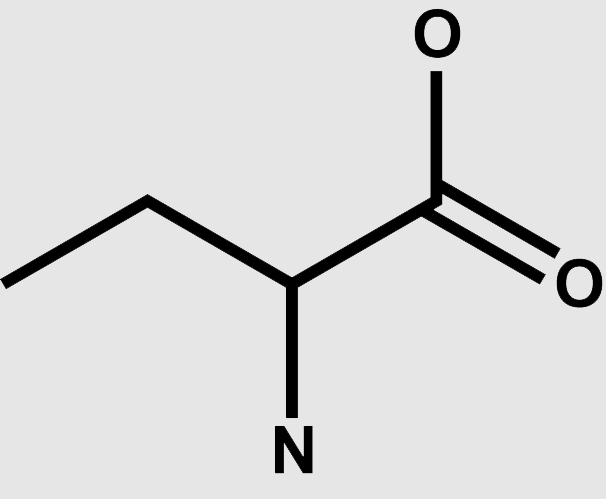 CCC(N)C(O)=O |
| [Metallo-dependent hydrolases](http://scop.mrc-lmb.cam.ac.uk/scop/data/scop.b.d.b.ba.html) |  | c.1.9 |  | 48 |  | 8 |  | [x.x.x.x](http://www.chem.qmul.ac.uk/iubmb/enzyme/) |  | - |
|  |  |  |  |  |  |  |  |  |  |  |
