# Supplementary material for: Evolutionarily Conserved Substrate Substructures for Automated Annotation of Enzyme Superfamilies
Source: PLoS Comput Biol. 2008 Aug 1;4(8):e1000142. doi: 10.1371/journal.pcbi.1000142 (PMC2453236; doi:10.1371/journal.pcbi.1000142)
Supplement: Table S2 — Overlap between reacting and conserved substructures (fc and fr). The superfamilies in this table are sorted by [average fc(atoms) plus fc(bonds)]. *The metallo-dependent hydrolases superfamily does not have a substrate substructure that is conserved in all members of the superfamily. Thus, for this superfamily, fc, the fraction of the conserved substructure that is reacting, cannot be calculated. (0.16 MB DOC) [file pcbi.1000142.s002.doc]

|  | | | | | | | | | | | | | | |
| --- | --- | --- | --- | --- | --- | --- | --- | --- | --- | --- | --- | --- | --- | --- |
|  | | | | | | | | | | | | | | |
|  |  |  |  | **f_c_** | | | | |  | **f_r_** | | | | |
|  |  |  |  | **Avg.** | |  | **Std. Dev.** | |  | **Avg.** | |  | **Std. Dev.** | |
| **Superfamily** |  | **SCOP ID** |  | **Atoms** | **Bonds** |  | **Atoms** | **Bonds** |  | **Atoms** | **Bonds** |  | **Atoms** | **Bonds** |
|  |  |  |  |  |  |  |  |  |  |  |  |  |  |  |
| Alkaline phosphatase-like |  | c.76.1 |  | 0.98 | 0.98 |  | 0.04 | 0.05 |  | 0.66 | 0.64 |  | 0.06 | 0.05 |
| SGNH hydrolase |  | c.23.10 |  | 0.95 | 0.92 |  | 0.02 | 0.03 |  | 0.65 | 0.55 |  | 0.04 | 0.01 |
| Nitrile hydratase alpha chain |  | d.159.1 |  | 0.72 | 0.68 |  | 0.16 | 0.17 |  | 0.82 | 0.81 |  | 0.26 | 0.27 |
| Carbohydrate phosphatase |  | e.7.1 |  | 0.72 | 0.67 |  | 0.01 | 0 |  | 0.98 | 0.97 |  | 0.03 | 0.03 |
| Cobalamin (vitamin B12)-dependent enzymes |  | c.1.19 |  | 0.81 | 0.54 |  | 0.09 | 0.06 |  | 0.81 | 0.81 |  | 0.09 | 0.09 |
| Phosphoglycerate mutase-like |  | c.60.1 |  | 0.61 | 0.55 |  | 0.29 | 0.32 |  | 0.68 | 0.64 |  | 0.31 | 0.38 |
| Six-hairpin glycosidases |  | a.102.1 |  | 0.56 | 0.48 |  | 0.14 | 0.15 |  | 0.65 | 0.67 |  | 0.13 | 0.18 |
| alpha/beta-Hydrolases |  | c.23.9 |  | 0.55 | 0.48 |  | 0.23 | 0.21 |  | 0.64 | 0.63 |  | 0.45 | 0.47 |
| PLP-binding barrel |  | c.1.6 |  | 0.56 | 0.47 |  | 0.1 | 0.12 |  | 1 | 1 |  | 0 | 0 |
| Carbon-nitrogen hydrolase |  | d.160.1 |  | 0.5 | 0.5 |  | 0.71 | 0.71 |  | 0.07 | 0.04 |  | 0.09 | 0.06 |
| Creatinase/aminopeptidase |  | d.127.1 |  | 0.55 | 0.44 |  | 0.24 | 0.3 |  | 0.56 | 0.45 |  | 0.23 | 0.31 |
| Metalloproteases ("zincins"), catalytic domain |  | d.92.1 |  | 0.55 | 0.43 |  | 0.31 | 0.38 |  | 0.28 | 0.13 |  | 0.17 | 0.14 |
| Nudix |  | d.113.1 |  | 0.5 | 0.46 |  | 0.2 | 0.22 |  | 0.46 | 0.40 |  | 0.2 | 0.18 |
| Phospholipase C/P1 nuclease |  | a.124.1 |  | 0.5 | 0.43 |  | 0 | 0 |  | 0.52 | 0.48 |  | 0.21 | 0.22 |
| Pyruvoyl-dependent histidine and arginine decarboxylases |  | d.155.1 |  | 0.47 | 0.38 |  | 0.06 | 0.07 |  | 0.93 | 0.95 |  | 0.09 | 0.07 |
| PLC-like phosphodiesterases |  | c.1.18 |  | 0.43 | 0.36 |  | 0.15 | 0.16 |  | 0.45 | 0.39 |  | 0.1 | 0.07 |
| dUTPase-like |  | b.85.4 |  | 0.41 | 0.35 |  | 0.03 | 0 |  | 0.92 | 0.9 |  | 0.12 | 0.14 |
| Tautomerase/MIF |  | d.80.1 |  | 0.58 | 0.17 |  | 0.14 | 0.17 |  | 0.32 | 0.13 |  | 0.16 | 0.13 |
| Xyloase isomerase-like |  | c.1.15 |  | 0.43 | 0.23 |  | 0.26 | 0.24 |  | 0.71 | 0.49 |  | 0.19 | 0.24 |
| Zn-dependent exopeptidases |  | c.56.5 |  | 0.42 | 0.19 |  | 0.18 | 0.13 |  | 0.37 | 0.07 |  | 0.16 | 0.04 |
| Chelatase |  | c.92.1 |  | 0.33 | 0.23 |  | 0.09 | 0.08 |  | 0.68 | 0.47 |  | 0.02 | 0.11 |
| L-aspartase-like |  | a.127.1 |  | 0.38 | 0.17 |  | 0.31 | 0.41 |  | 0.41 | 0.1 |  | 0.13 | 0.24 |
| Protease propeptides/inhibitors |  | d.58.3 |  | 0.30 | 0.14 |  | 0.05 | 0.13 |  | 0.62 | 0.47 |  | 0.21 | 0.42 |
| Ribulose-phosphate binding barrel |  | c.1.2 |  | 0.28 | 0.13 |  | 0.28 | 0.13 |  | 0.37 | 0.25 |  | 0.36 | 0.25 |
| Metallo-hydrolase/oxidoreductase |  | d.157.1 |  | 0.25 | 0.15 |  | 0.15 | 0.11 |  | 0.92 | 0.88 |  | 0.12 | 0.18 |
| Enolase C-terminal domain-like |  | c.1.11 |  | 0.31 | 0.08 |  | 0.1 | 0.13 |  | 0.35 | 0.07 |  | 0.07 | 0.12 |
| Thioesterase/thiol ester dehydrase-isomerase |  | d.38.1 |  | 0.19 | 0.19 |  | 0.34 | 0.33 |  | 0.12 | 0.11 |  | 0.21 | 0.18 |
| Cobalamin (vitamin B12)-binding domain |  | c.23.6 |  | 0.21 | 0.17 |  | 0.30 | 0.26 |  | 0.38 | 0.33 |  | 0.53 | 0.47 |
| Subtilisin-like |  | c.41.1 |  | 0.23 | 0.14 |  | 0.09 | 0.06 |  | 0.57 | 0.52 |  | 0.36 | 0.42 |
| Kringle-like |  | g.14.1 |  | 0.22 | 0.13 |  | 0.24 | 0.25 |  | 0.3 | 0.11 |  | 0.19 | 0.21 |
| beta-lactamase/ transpeptidase-like |  | e.3.1 |  | 0.29 | 0.06 |  | 0.26 | 0.1 |  | 0.28 | 0.03 |  | 0.26 | 0.05 |
| (Phosphotyrosine protein) phosphatases II |  | c.45.1 |  | 0.2 | 0.13 |  | 0.19 | 0.18 |  | 0.07 | 0.04 |  | 0.06 | 0.05 |
| FAH |  | d.177.1 |  | 0.22 | 0.07 |  | 0.1 | 0.12 |  | 0.34 | 0.04 |  | 0.15 | 0.07 |
| HD-domain/PDEase-like |  | a.211.1 |  | 0.17 | 0.08 |  | 0 | 0 |  | 0.79 | 0.81 |  | 0.02 | 0.04 |
| Cytidine deaminase-like |  | c.97.1 |  | 0.20 | 0 |  | 0.05 | 0 |  | 0.26 | 0 |  | 0.07 | 0 |
| Isochorismatase-like hydrolases |  | c.33.1 |  | 0.17 | 0 |  | 0.24 | 0 |  | 0.25 | 0 |  | 0.35 | 0 |
| Glutaminase/Asparaginase |  | c.88.1 |  | 0.13 | 0.03 |  | 0.06 | 0.04 |  | 0.35 | 0.11 |  | 0.14 | 0.15 |
| Caspase-like |  | c.17.1 |  | 0.11 | 0.03 |  | 0.08 | 0.04 |  | 0.33 | 0.11 |  | 0.11 | 0.16 |
| AraD-like aldolase/epimerase |  | c.74.1 |  | 0.12 | 0.01 |  | 0.02 | 0.01 |  | 0.38 | 0.01 |  | 0.16 | 0.02 |
| EGF/Laminin |  | g.3.11 |  | 0.04 | 0 |  | 0.04 | 0 |  | 0.06 | 0 |  | 0.06 | 0 |
| Arginase/deacetylase |  | c.42.1 |  | 0.04 | 0 |  | 0.05 | 0 |  | 0.06 | 0 |  | 0.09 | 0 |
| *Metallo-dependent hydrolases |  | c.1.9 |  | -- | -- |  | -- | -- |  | 0 | 0 |  | 0 | 0 |
|  |  |  |  |  |  |  |  |  |  |  |  |  |  |  |
