# Supplementary material for: Evolutionarily Conserved Substrate Substructures for Automated Annotation of Enzyme Superfamilies
Source: PLoS Comput Biol. 2008 Aug 1;4(8):e1000142. doi: 10.1371/journal.pcbi.1000142 (PMC2453236; doi:10.1371/journal.pcbi.1000142)
Supplement: Table S3 — Superfamilies annotation list. These structures are mostly from structural genomics projects. Annotation of these superfamilies with their conserved substructures may provide useful guidance for analyses to determine the function of these proteins or to identify characteristics of ligands useful for co-crystallization attempts. (0.14 MB DOC) [file pcbi.1000142.s003.doc]

|  | | | |
| --- | --- | --- | --- |
|  | | | |
| **Superfamily** | **SCOP ID** | **PDB ID** | **Current Annotation in PDB** |
|  |  |  |  |
| alpha/beta-Hydrolases | c.69.1 | 1vkh | Putative serine hydrolase Ydr428c |
|  |  | 1pv1 | Hypothetical esterase YJL068C |
|  |  | 1r3d | Hypothetical protein VC1974 |
| AraD-like aldolase/epimerase | c.74.1 | 1pvt | Putative sugar-phosphate aldolase |
| Arginase/deacetylase | c.42.1 | 1c3p, 1c3r, 1c3s | HDAC homologue |
| beta-lactamase/ transpeptidase-like | e.3.1 | 1mki, 2osu | Probable glutaminase YbgJ |
|  |  | 1u60 | Probable glutaminase YbaS |
| Carbohydrate phosphatase | e.7.1 | 1ni9 | Glycerol metabolism protein GlpX |
| Carbon-nitrogen hydrolase | d.160.1 | 1f89 | hypothetical protein yl85 |
|  |  | 1j31 | Hypothetical protein PH0642 |
| Cytidine deaminase-like | c.97.1 | 2g84 | Putative deaminase NE0047 |
|  |  | 1vk9 | Hypothetical protein TM1506 |
| Enolase C-terminal domain-like | c.1.11 | 1rvk | Hypothetical protein Atu3453 |
|  |  | 1zz, 2dw6, 2dw7 | Hypothetical protein Bll6730 |
|  |  | 2gdq, 2gge, | Hypothetical protein YitF |
|  |  | 2gl5 | Putative dehydratase protein STM2273 |
| FAH | d.177.1 | 1nr9 | Putative isomerase YcgM |
|  |  | 1nkq | Hypothetical protein Ynl168c |
| HD-domain/PDEase-like | a.211.1 | 1ynb, 1yoy | Hypothetical protein AF1432 |
|  |  | 2hek | Hypothetical protein aq_1910 |
|  |  | 2o6i | Hypothetical protein EF1143 |
|  |  | 1vqr | Hypothetical protein Cj0248 |
| Isochorismatase-like hydrolases | c.33.1 | 1yac | YcaC |
|  |  | 1j2r | Hypothetical protein YecD |
| Metallo-dependent phosphatases | d.159.1 | 1nmw | Hypothetical protein PF1291 |
|  |  | 1uf3 | Hypothetical protein TT1561 |
|  |  | 1s3m, 1s3l, 1s3n, 2ahd | Putative phosphodiesterase MJ0936 |
|  |  | 1xm7 | Hypothetical protein aq_1666 |
|  |  | 1t70 | Putative phosphatase DR1281 |
|  |  | 1t71 | Hypothetical protein MPN349 |
|  |  | 2cv9 | Hypothetical protein TTHA0625 |
| Metallo-hydrolase/oxidoreductase | d.157.1 | 1vjn | Hypothetical protein TM0207 |
|  |  | 1zkp | Hypothetical protein BA1088 (BAS1016) |
|  |  | 2az4 | Hypothetical protein EF2904 |
|  |  | 1ztc | Hypothetical protein TM0894 |
| Metalloproteases ("zincins"), catalytic domain | d.92.1 | 1oz9 | Hypothetical protein Aq_1354 |
|  |  | 1xm5 | Hypothetical protein YbeY |
|  |  | 1tvi | Hypothetical protein TM1509 |
| Nudix | d.113.1 | 1sjy, 1sz3, 1su2, 1soi | Hypothetical protein DR1025 |
|  |  | 1k2e, 1k26, 1jrk | Hypothetical protein PAE3301 |
|  |  | 2azw | Hypothetical protein EF1141 |
|  |  | 2b0v | Hypothetical protein NE0184 |
|  |  | 2b06 | Hypothetical protein SP1235 (spr1115) |
|  |  | 1q27 | Hypothetical protein DR0079 |
|  |  | 2fkb | Hypothetical protein YfcD |
|  |  | 2fml | Hypothetical protein EF2700, N-term. domain |
|  |  | 2fbl | Hypothetical protein BT0354, N-term. domain |
| (Phosphotyrosine protein) phosphatases II | c.45.1 | 1xri, 2q47 | Putative phosphatase At1g05000 |
| PLC-like phosphodiesterases | c.1.18 | 1o1z | Hypothetical protein TM1621 |
|  |  | 1vd6, 1v8e | Putative glycerophosphodiester PDE TTHB141 |
| PLP-binding barrel | c.1.6 | 1ct5, 1b54 | "Hypothetical" protein ybl036c |
| Ribulose-phosphate binding barrel | c.1.2 | 1y0e | Putative NanE |
|  |  | 1yxy | Putative NanE |
| SGNH hydrolase | c.23.10 | 1z8h, 1vjg | Hypothetical protein alr1529 (putative lipase) |
|  |  | 2apj | Putative acetylxylan esterase At4g34215 |
| Six-hairpin glycosidases | a.102.1 | 1wzz | Probable endoglucanase CmcAX |
|  |  | 2afa | Putative NAG isomerase YihS |
|  |  | 1nc5, 2d8l | Hypothetical protein YteR |
| Tautomerase/MIF | d.80.1 | 1mww | Hypothetical protein HI1388.1 |
|  |  | 1u9d | Hypothetical protein VC0714 |
| Thioesterase/thiol ester dehydrase-isomerase | d.38.1 | 1njk | Hypothetical protein YbaW |
|  |  | 1s5u | Hypothetical protein YbgC |
|  |  | 1yli | Putative acyl-coa thioester hydrolase HI0827 |
|  |  | 2hlj | Hypothetical protein PP0301 |
|  |  | 2fuj | Hypothetical protein XCC1147 |
|  |  | 2gvh | Probable acyl-CoA hydrolase AGR_L_2016 |
|  |  | 2cye | Probable thioesterase TTHA1846 |
|  |  | 1z54 | Probable thioesterase TTHA0908 |
|  |  | 2nuj | Hypothetical protein Jann_1972 |
|  |  | 2gf6 | Hypothetical protein SSO2295 |
|  |  | 2av9 | Hypothetical thioesterase PA5185 |
|  |  | 2hx5 | Hypothetical protein PMT2055 |
|  |  | 2ali | Hypothetical protein PA2801 |
|  |  | 2bi0 | Hypothetical protein Rv0216/MT0226 |
|  |  | 2b3n, 2b3m | Hypothetical protein AF1124 |
|  |  | 2c2i | Hypothetical protein Rv0130 |
|  |  | 1sc0, 1o0i, 2b6e | Hypothetical protein HI1161 |
|  |  | 1vh5, 1sbk, 1vi8 | Hypothetical protein YdiI |
|  |  | 1vh9 | Hypothetical protein YbdB |
|  |  | 1ixl | Hypothetical protein PH1136 |
|  |  | 1t82 | Putative thioesterase SO4397 |
|  |  | 1sh8 | Hypothetical protein PA5026 |
|  |  | 1zki | Hypothetical protein PA5202 |
|  |  | 2hbo | Hypothetical protein CC3309 |
|  |  | 2cy9 | Hypothetical protein Them2 |
|  |  | 2h4u, 2f0x | Hypothetical protein Them2 |
|  |  | 1yoc | Hypothetical protein PA1835 |
|  |  | 2cwz | Hypothetical protein TTHA0967 |
| Xylose isomerase-like | c.1.15 | 1i60, 1i6n | Hypothetical protein IolI |
|  |  | 2g0w | Hypothetical protein Lmo2234 |
|  |  | 1k77 | Hypothetical protein YgbM (EC1530) |
|  |  | 1yx1 | Hypothetical protein PA2260 |
| Zn-dependent exopeptidases | c.56.5 | 1vhe | Hypothetical protein YsdC, catalytic domain |
|  |  | 1vho | Putative endoglucanase TM1048, cat. domain |
|  |  | 1y7e | Probable aminopeptidase ApeA |
|  |  |  |  |
